# Orthoceratoid and coleoid cephalopods from the Middle Triassic of Switzerland with an updated taxonomic framework for Triassic Orthoceratoidea

**DOI:** 10.1186/s13358-024-00307-8

**Published:** 2024-04-03

**Authors:** Alexander Pohle, Christian Klug

**Affiliations:** 1 Institut für Geologie, Mineralogie und Geophysik, Ruhr-Universität Bochum, 44801 Bochum, Germany; 2 Paläontologisches Institut und Museum, Universität Zürich, Karl-Schmid-Strasse 4, 8006 Zurich, Switzerland

**Keywords:** Monte San Giorgio, Anisian, Cephalopoda, Nautiloids, Orthocone, Pseudorthocerida, Orthocerida, Trematoceratidae, Coleoidea

## Abstract

Orthoconic cephalopods are subordinate, but persistent, widespread and regionally abundant components of Triassic marine ecosystems. Here, we describe unpublished specimens from the Anisian (Middle Triassic) Besano Formation at Monte San Giorgio, Switzerland. They can be assigned to two major but unrelated lineages, the Coleoidea and the Orthoceratoidea. The orthoceratoids belong to *Trematoceras elegans* (Münster, 1841) and occur regularly within the Besano Formation, are uniform in size, and have few available morphological characters. In contrast, coleoids are more diverse and appear to be restricted to shorter intervals. A new coleoid is described as *Ticinoteuthis chuchichaeschtli* gen. et sp. nov. To better put the orthoceratoids of the Besano Formation into perspective, we also synthesise the current taxonomy of Triassic orthoceratoids on a global scale. The currently used scheme is largely outdated, with very little taxonomic progress in the past 100 years. Despite previous research showing the distinctness of Triassic orthoceratoids from Palaeozoic taxa, they are still commonly labelled as “*Orthoceras*” or “*Michelinoceras*”, which are confined to the Palaeozoic. We show that Triassic orthoceratoids probably belong to a single lineage, the Trematoceratidae, which can be assigned to the Pseudorthocerida based on the embryonic shell and endosiphuncular deposits. Many Triassic species can probably be assigned to *Trematoceras*, but there are at least two additional Triassic orthoceratoid genera, *Paratrematoceras* and *Pseudotemperoceras*. Finally, we review the palaeobiogeographic and stratigraphic distribution of the group and outline possible future research directions.

## Introduction

Cephalopods with straight (orthoconic) conchs represent a heterogeneous group that evolved repeatedly in different lineages of cephalopods. A large proportion of orthocones can be assigned to the subclass Orthoceratoidea (Kröger, 2008; Pohle et al., 2022), although this conch shape evolved independently within the Multiceratoidea and Endoceratoidea (Evans & King, 2012; Pohle et al., 2022), as well as the Ammonoida (Hoffmann et al., 2021), while the Bactritoida and early Coleoidea retained this conch shape as a plesiomorphy (Klug et al., 2019). Orthoceratoids were common during the early Palaeozoic with frequent mass occurrences (e.g., Bogolepova & Holland, 1995; Hewitt & Watkins, 1980; Kröger & Pohle, 2021; Pohle & Klug, 2018a), but their diversity declined during the late Palaeozoic, until they went extinct in the Late Triassic (Sweet, 1964). The subclass is generally poorly studied, partly because of the lack of diagnostic characters, frequent homeomorphies and the fact that any meaningful systematic treatment requires sectioning of fossils to investigate internal structures (Flower, 1962; Kröger, 2008). At the same time, orthoceratoids represent an evolutionary important group, as they contain the probable ancestors of both ammonoids and coleoids via the Bactritoida, and perhaps also of the Nautiloidea sensu stricto (Klug et al., 2015a, 2019; Kröger et al., 2011; Pohle et al., 2022).

Early coleoids share a longiconic orthoconic phragmocone with orthoceratoids and bactritoids, leading sometimes to difficulties in distinguishing between the two groups where they overlap stratigraphically (Flower & Gordon, 1959; Fuchs, 2021; Mapes, 1979; Mapes et al., 2010) and where the mineralised hard parts are not preserved. This problem is less evident in the Triassic due to the demise of the bactritids at the end of the Permian (Erben, 1964). However, while a rostrum unequivocally characterises a coleoid, many Triassic species are still only known from phragmocones (Fuchs, 2021; Mariotti et al., 2021). Consequently, if only phragmocones are available, a ventral siphuncle unambiguously identifies a coleoid in Triassic orthocones (Jeletzky, 1966), while a central siphuncle is restricted to orthoceratoids (Schastlivceva, 1988). Poor preservation can thus still present challenges if the position of the siphuncle remains unclear, especially considering the generally longiconic shape of the phragmocone of aulacoceratids (Mariotti et al., 2021). These difficulties are also demonstrated by the fact that multiple Triassic aulacoceratid genera are based on species originally described in the nineteenth century as *“Orthoceras”* or *“Orthoceratites”* (Mariotti et al., 2021). Despite this potential for misidentifications, Triassic orthoceratoids and coleoids are only rarely directly compared to each other for taxonomic purposes. In general, Triassic members of both groups are poorly studied.

To contribute to the knowledge on Triassic orthoconic cephalopods, we here describe previously undocumented occurrences of orthoconic cephalopods from the Besano Formation (late Anisian, Middle Triassic) of the Monte San Giorgio UNESCO World Heritage site in Ticino, Switzerland. The cephalopods from this locality have been described extensively by Rieber (1970, 1973, 1974), including mostly ammonoids, but also some important findings of coleoids and several isolated orthoceratoids. Nautilids are exceedingly rare in the Besano formation and have only been described recently (Pieroni, 2022). Since cephalopods are typically preserved only as external moulds in the Besano Formation, they have not been collected extensively, especially in the case of coleoids and orthoceratoids.

While the material reported here does not allow for a detailed taxonomic treatment, we show their stratigraphic distribution and variation in size, extending their previously known range in the Besano Formation, although the limited amount of data does not provide evidence for multiple species of orthoceratoids. In contrast, the coleoids appear to be more variable and more restricted to short time intervals. Among them are several specimens with small apical angles and depressed cross section unlike any other known Triassic coleoid, which we assign to a new taxon. We also discuss the taxonomic status of Triassic orthoceratoids more generally, which are still commonly labelled as “*Orthoceras*” or “*Michelinoceras*”; these names, however, refer to exclusively Palaeozoic genera. A thorough revision of Triassic orthoceratoids is needed but out of the scope of this study, as it would require finding and revisiting original material of a large number of previously described species, many with uncertain repositories of the type material. Instead, we provide an overview of the current state of the taxonomy and stabilise it in a provisional classification scheme that reflects updated understanding of fossil cephalopod systematics. This overview will serve as a useful guide for any future revisions or taxonomic treatments of the group.

## Material and methods

All fossils described here are from the Middle Triassic Besano Formation of Monte San Giorgio. In particular, they were collected from strata of the Grenzbitumenzone, which has been dated to an age between 240.63 ± 0.13 and 241.07 ± 0.13 million years (Stockar et al., 2012). These strata were mined for the bituminous sediments from 1906 until 1950 (Lanz & Felber, 2020). The strata consist of dolomites and black shales of varying composition. Röhl et al., (2001, fig. 3) documented that laminated mud-, wacke- and packstones dominate, which alternate with organic-rich marl and claystone as well as microbialites and subordinate dolomitic wacke- and packstones layers. According to their analyses (Röhl et al., 2001, fig. 2), the carbonate content mostly lies between 75 and 100% in the lower and upper Grenzbitumenzone, while the middle Grenzbitumenzone has frequent layers with much lower carbonate content. This part of the succession with reduced carbonate content yielded most of the non-ammonoid cephalopods. This suggests that cephalopods occurred mostly during times of high sea-levels and when more or less pelagic conditions prevailed. Remarkably, many of the specimens discussed here come from the more carbonatic layers.

We investigated the orthoconic cephalopods from the Monte San Giorgio housed in the collection of the Palaeontological Institute and Museum of the University of Zurich (PIMUZ). This material has been collected during the past 100 years in various excavations and field trips led by the University of Zurich, though without being a major focus due to the poor to moderately good preservation and low morphological variability. Out of the 43 specimens not mentioned by Rieber (1973), 22 were preserved as external moulds, the rest retained at least part of the phragmocone as internal moulds. We recorded the stratigraphic position of all orthoconic cephalopods within the Besano Formation and additionally measured their maximum and minimum diameters as well as the specimen lengths to calculate apical angles following Pohle and Klug (2018a). These values were compared across the stratigraphy to investigate morphological patterns. Furthermore, we consulted an unpublished systematic list of specimens collected at the locality Point 902/Mirigioli (see Röhl et al., 2001, fig. 1), generously provided by H. Furrer (Zurich). This list provided additional information on the stratigraphic distribution and relative abundance of orthoconic cephalopods in the Besano Formation. Note that the terms orthoconic, longiconic and breviconic have slightly different historical meanings for coleoids and orthoceratoids. In coleoids, each term refers to a specific range of apical angles (e.g., Fuchs, 2021), while orthoconic refers to the straight shape of the conch independently of the apical angle in orthoceratoids (Teichert, 1964). Thus, low-angled orthocones are longiconic, while high-angled orthocones are breviconic, though an exact cut-off angle has never been defined. We use the latter definition, although we supply the values of apical angles directly, where relevant.

Institutional abbreviations: PIMUZ, Paläontologisches Institut und Museum, University of Zurich, Switzerland; SNSB-BSPG, Staatliche Naturwissenschaftliche Sammlungen Bayerns—Bayerische Staatssammlung für Paläontologie und Geologie, Munich, Germany.

## History of research on Triassic orthoceratoids

Since the middle of the nineteenth century, at least 53 species of Triassic orthoceratoids have been named worldwide and described in varying levels of detail (not counting species that were initially described as “*Orthoceras*” but later identified as coleoid phragmocones; Table 1). More than half of these orthoceratoids were described prior to 1930, mostly in German, and never underwent a modern revision (Bülow, 1915; d’Orbigny, 1850; Diener, 1907; Frech, 1907; Gabb, 1864; Gemmellaro, 1904; Hauer, 1846, 1847, 1849, 1888; Hyatt & Smith, 1905; Kittl, 1908; Klipstein, 1843; Kutassy, 1927; Mojsisovics, 1869, 1873; Münster, 1841; Reis, 1901; Stoppani, 1859). Accordingly, many species are known exclusively from drawings, with uncertain repository of the type material, if a type has been selected at all. All of these species of Triassic orthoceratoids were assigned to the genus *Orthoceras* (or its alternative, now obsolete spelling *Orthoceratites*), a genus which has its own very complicated taxonomic history. It has long been established that “true” *Orthoceras* is restricted to a group of species not known outside of the Darriwilian (Middle Ordovician) of Baltoscandia and corresponding erratics, based on the type species *Orthoceras regulare* Schlotheim, 1820 (ICZN, 1974; Kröger, 2004; Melville, 1959, 1970; Teichert & Miller, 1936; Troedsson, 1931). This species is unique among orthoceratoids due to its bizarre, prominent longitudinal impressions on the body chamber (Kröger, 2004; Sweet, 1964; Troedsson, 1931), meaning that all orthoceratoids lacking this feature must be assigned to another genus. Despite this, the name *Orthoceras* is still commonly used erroneously for orthoconic cephalopods outside of this definition, particularly in Museum exhibitions and commercially traded specimens. Thus, it is not surprising that the taxon name *Orthoceras* is also widespread in the present-day scientific literature as a taxonomic identifier for Triassic orthoceratoids. In our opinion, continuing this practice is akin to calling every unidentifiable bipedal non-avian dinosaur fossil “*Tyrannosaurus*”, a solution that nobody would seriously consider. Consequently, we here urge the scientific community to, when in doubt, use names in open nomenclature instead, such as Orthocerida indet., Pseudorthocerida indet., or Orthoceratoidea indet (see also King & Evans, 2019; Pohle et al., 2022 for phylogenetic context and discussions of endings of higher-level taxonomic names).

**Table 1:** List of all previously described Triassic orthoceratoid species

Species	Age of type	Type area
*Trematoceras elegans* (Münster, 1841)	Carnian	Dolomites
*T. boreale* Schastlivceva, 1981	Olenekian	West Verkhoyansk
*T. campanile* (Mojsisovics, 1869)^1^	Anisian	Salzkammergut
*T. clarum* Schastlivceva, 1986	Olenekian	West Verkhoyansk
*T. dubium* (Hauer, 1847)^1^	Anisian	Salzkammergut
*T. freieslebense* (Klipstein, 1843)^1^	Carnian	Dolomites
*T. hikichii* Niko et al., 2016	Olenekian	Miyagi
*T. insperatum* Schastlivceva, 1988	Anisian	North Caucasus
*T. lytosiphon* (Gemmellaro, 1904)	Norian	Sicily
*T. mangishlakense* Schastlivceva, 1981	Olenekian	Mangyshlak
*T. politum* (Klipstein, 1843)^1^	Carnian	Dolomites
*T. solidum* Schastlivceva, 1988	Olenekian	Mangyshlak
*T. subcampanile* Kiparisova, 1954	Olenekian	South Primorye
*T. vulgare* Schastlivceva, 1981	Olenekian	Mangyshlak
*T. watanabei* Niko & Ehiro, 2020	Anisian	Miyagy
“*T. memorabile*” Grădinaru et al., 2007^2^	Olenekian	North Dobrogea
“*T. moderatum*” Grădinaru et al., 2007^2^	Anisian	North Dobrogea
“*T. potissimum*” Grădinaru et al., 2007^2^	Olenekian	North Dobrogea
*Paratrematoceras shevyrevi* Schastlivceva, 1981	Anisian	North Caucasus
*Pa. ornatum* Schastlivceva, 1981	Anisian	North Caucasus
“*Pa. abundans*” Grădinaru et al., 2007^2^	Olenekian	North Dobrogea
“*Pa. conspicuum*” Grădinaru et al., 2007^2^	Anisian	North Dobrogea
“*Pa. productum*” Grădinaru et al., 2007^2^	Anisian	North Dobrogea
*Phatthalungoceras srisuki* Tongtherm & Nabhitabhata, 2018	Anisian	Peninsular Thailand
*Pseudotemperoceras pulchrum* Schastlivceva, 1986	Olenekian	West Verkhoyansk
*Ps. nyalamense* (Chen, 1981)	Olenekian	Tulong
“*Romanorthoceras resupinum*” Grădinaru et al., 2007^2^	Olenekian	North Dobrogea
“*Orthoceras*” *acum* Reis, 1907^3^	Anisian	Wetterstein
“*O.*” *austriacum* Mojsisovics, 1873	Norian	Salzkammergut
“*O.*” *baconicum* Frech, 1907	Ladinian	Bakony
“*O.*” *billiemense* Gemmellaro, 1904	Norian	Sicily
“*O.*” *blakei* (Gabb, 1864)^4^	Anisian	Humboldt Range
“*O.*” *celticum* Mojsisovics, 1873	Carnian	Graz Highlands
“*O.*” *increscens* Kittl, 1908	Ladinian	North Dobrogea
“*O.*” *indoaustralicum* Bülow, 1915	Late Triassic	Timor
“*O.*” *lateseptatum* Hauer, 1846	Anisian	Salzkammergut
“*O.*” *lennaensis* (Stoppani, 1859)^4^	Ladinian	Dolomites
“*O.*” *mojsisovicsi* Salomon, 1895^5^	Anisian	Dolomites
“*O.*” *multilabiatum* Hauer, 1888	Anisian	Sarajevo
“*O.*” *nodosum* Kutassy, 1927	Carnian	Budapest
“*O.*” *politum acumitatum* Leonardi & Polo, 1952	Ladinian	Dolomites
“*O.*” *pulchellum* Hauer, 1849	Late Triassic	Salzkammergut
“*O.*” *pulchristriatum* Bülow, 1915	Late Triassic	Timor
“*O.*” *rotundulum* Bülow, 1915	Anisian	Timor
“*O.*” *salinarium* Hauer, 1846	Norian	Salzkammergut
“*O.*” *sandlingense* Mojsisovics, 1873	Carnian	Graz Highlands
“*O.*” *shastense* Hyatt & Smith, 1905	Carnian	Cascade Range
“*O.*” *spitiense* Diener, 1907	Ladinian	Spiti
“*O.*” *styriacum* Mojsisovics, 1873^6^	Carnian	Graz Highlands
“*O.*” *subellipticum* d’Orbigny, 1850^6^	Carnian	Dolomites
“*O.*” *subtiliseptatum* Gemmellaro, 1904	Norian	Sicily
“*O.*” *triadicum* Mojsisovics, 1873	Ladinian	Graz Highlands
“*O.*” *variestriatum* Reis, 1901	Anisian	Wetterstein

Note that we only assign species with known internal characters to genera. Consequently, many species are here still listed as “*Orthoceras*”, even if this designation is incorrect. These species are provisionally considered nomina dubia

^1^Likely synonym of *T. elegans*, see systematic palaeontology section

^2^As Grădinaru et al. (2007) did not provide any descriptions nor illustrations, these taxa must be considered nomina nuda

^3^Preoccupied by *O. acus* Roemer, 1855 and *O. acus* Barrande, 1868

^4^Originally described under the (now unavailable) genus *Orthoceratites*

^5^Preoccupied by *O. mojsisovicsi* Barrande, 1877

^6^Potential coleoid phragmocone

If *Orthoceras* is an outdated name for these taxa, to what genus should the species be assigned instead? A solution to this problem was proposed almost a century ago: as a replacement for taxa that could no longer be accommodated within *Orthoceras*, Foerste (1932) established the genus *Michelinoceras* with the late Silurian *Michelinoceras michelini* (Barrande, 1866) as type species. Subsequently, *Michelinoceras* became a waste basket taxon for inconspicuous orthoceratoids with smooth shells, low apical angle, central siphuncles and wide cameral spacing that occur from the Ordovician up to the Triassic, effectively taking over the previous role of *Orthoceras* (Sweet, 1964). Although it was already mentioned sixty years ago that Carboniferous to Triassic species were probably unrelated to the type species (Sweet, 1964), the practice to refer to Triassic orthoceratoids as *Michelinoceras* as an alternative to *Orthoceras* continued (e.g., Chen, 1981; Rieber, 1973; Silberling & Nichols, 1982; Wang et al., 2008).

Despite this confusion and prevalent use of waste basket taxa, there was already an available genus name to refer to Triassic orthoceratoids since the middle of the nineteenth century: *Trematoceras* Eichwald, 1851 with the type species *Trematoceras elegans* (Münster, 1841). Although Eichwald (1851) based his genus on the misconception that it had a “disconnected” siphuncle when in fact the connecting rings were simply not preserved, his designation remains valid, even if his “*Trematoceras” discors* Eichwald, 1857 from the Ordovician of Estonia is unrelated and represents the type species of the poorly known *Balticoceras* Teichert, 1940. Eichwald (1851) therefore never conceived *Trematoceras* as a taxon restricted to the Triassic and the practice to assign species to *Orthoceras* remained until its narrower definition. Aside from the persistence of “*Orthoceras”* in the literature on Triassic cephalopods, attempts to group Triassic members of the genus were made by Mojsisovics (1873) and later Kutassy (1927) based on external characters of the shell. However, these groups of orthoceratoids were never applied more widely, partly because the distinction between smooth and striated shells is difficult in light of different preservational conditions and more importantly, because taxonomists realised the high relevance of internal structures for the classification of orthoconic nautiloids (e.g., Barrande 1865–1877; Hyatt, 1884; Flower, 1939). The genus *Trematoceras* was resurrected by Schindewolf (1933), who studied its embryonic shell, noting its similarity to *Pseudorthoceras* and distinctness from *Orthoceras*. According to him, all Triassic orthoceratoids probably belonged to the genus *Trematoceras*.

Ironically, this clarification of the taxonomic position of Triassic orthoceratoids was followed by a long dearth in taxonomic work on this group, with only one additional species and a new subspecies described within almost half a century (Kiparisova, 1954; Leonardi & Polo, 1952). Further mentions of Triassic orthoceratoids during this time came mostly in the form of faunal lists or brief descriptions, although this at least expanded the known palaeobiogeographic distribution of the group (e.g., Cecioni & Westermann, 1968; Creutzburg et al., 1966; Kummel & Erben, 1968). Jeletzky and Zapfe (1967) described additional specimens, one of which was suggested to be assigned to a new genus and family due to the presence of a coleoid-like sheath (i.e. primordial rostrum, see Fuchs, 2012). However, Jeletzky and Zapfe (1967) also noted that this feature may be pathological.

A short burst in research followed during the 1980s, where nine new Triassic species were described in two short papers and a monograph by Schastlivceva (1981, 1986, 1988). These studies (exclusively available in Russian language) still represent the most in-depth treatment of Triassic orthoceratoids to date. While most of Schastlivceva’s (1981, 1986, 1988) species were assigned to *Trematoceras*, she also established the new genera *Paratrematoceras* Schastlivceva, 1981 and *Pseudotemperoceras* Schastlivceva, 1986. Remarkably, despite all three genera falling into a relatively narrow morphological range of variation when compared to Palaeozoic orthoceratoids, she assigned each genus to a different family and in one case even to a different order: *Trematoceras* was placed in the Spyroceratinae of the Pseudorthoceratidae (Pseudorthocerataceae; now Pseudorthocerida), *Pseudotemperoceras* in the Geisonoceratidae (Orthocerataceae; now Orthocerida) and *Paratrematoceras* in the Michelinoceratinae of the Orthoceratidae (Orthocerataceae; now Orthocerida) (Schastlivceva, 1988). Although her studies represented a major step in the research of Triassic orthoceratoids, Schastlivceva’s studies mainly consisted in adding new species but did not contribute to revising the large number of already established species.

Only few further studies on Triassic orthoceratoids were published during the subsequent twenty years. Bizzarini and Gnoli (1991) revised cephalopods from the Cassian Formation of northern Italy, amongst others *Trematoceras elegans*, for which they provided modern photographs including SEM for the first time. Furthermore, they synonymised the second species from the Cassian Formation, *T. politum* with *T. elegans*. Sobolev (1994) summarised the stratigraphy of Siberian Triassic nautiloids, briefly mentioning some utility of orthoconic cephalopods in the stratigraphic zonation of the Olenekian. Another important study was published by Zakharov (1996) on the ultrastructure of the shell and septal necks, based on which he defined the Trematoceratidae to include all Triassic orthoceratoids. He assigned this family to the Pseudorthocerida, apparently independently of Schastlivceva (1981, 1986, 1988), which was not cited therein. The similarities between *Trematoceras* and *Pseudorthoceras* or other Pseudorthoceratidae with respect to their early ontogenetic stages and cameral deposits had already been noted by several other authors (Barskov, 1963; Dzik, 1984; Niko & Ehiro, 2020; Ristedt, 1968; Schindewolf, 1933; Teichert, 1940; Zhuravleva, 1978), though an alternative placement within the Orthoceratidae was still common (Balashov and Zhuravleva 1962; Sweet, 1964; Jeletzky & Zapfe, 1967; Bizzarini & Gnoli, 1991; Shigeta & Nguyen, 2014; Niko et al., 2016). However, it should be noted that the placement within the Orthocerida usually came without detailed justification. A pseudorthocerid affinity of *Trematoceras* was recently reaffirmed by Niko and Ehiro (2020) based on endosiphuncular deposits.

Recently, two new species of *Trematoceras* were described from Japan (Niko & Ehiro, 2020; Niko et al., 2016). While their species definitions involve clear diagnoses and adequate illustrations of the type material, comparisons with other species of *Trematoceras* are restricted to few species only, making differentiation between the species with overlapping definitions difficult. The new genus *Phatthalungoceras* was recently established based on material from Thailand (Tongtherm & Nabhitabhata, 2018; Tongtherm et al., 2016). However, the only known specimen of this genus is relatively poorly preserved and its validity difficult to assess without additional material. Further recent taxonomic names including several species and one new genus mentioned by Grădinaru et al. (2007) from eastern Romania must be considered as *nomina nuda* because they were not accompanied by diagnoses, descriptions or images.

In summary, research on Triassic orthoceratoids has a long history with phases of varying productivity and taxonomic concepts. Previous research almost entirely focused on describing new species, with a general revision of the group still pending. Most species are only known from fragmentary material, with either external or internal characters undocumented, and often unclear repository of the type material, if a type has been selected at all. With few exceptions, the studies refer to only a small part of the literature, i.e. mostly from the same region, despite the cosmopolitan distribution of the morphologically very similar taxa.

## Results

### Stratigraphic distribution

**Fig. 1:**
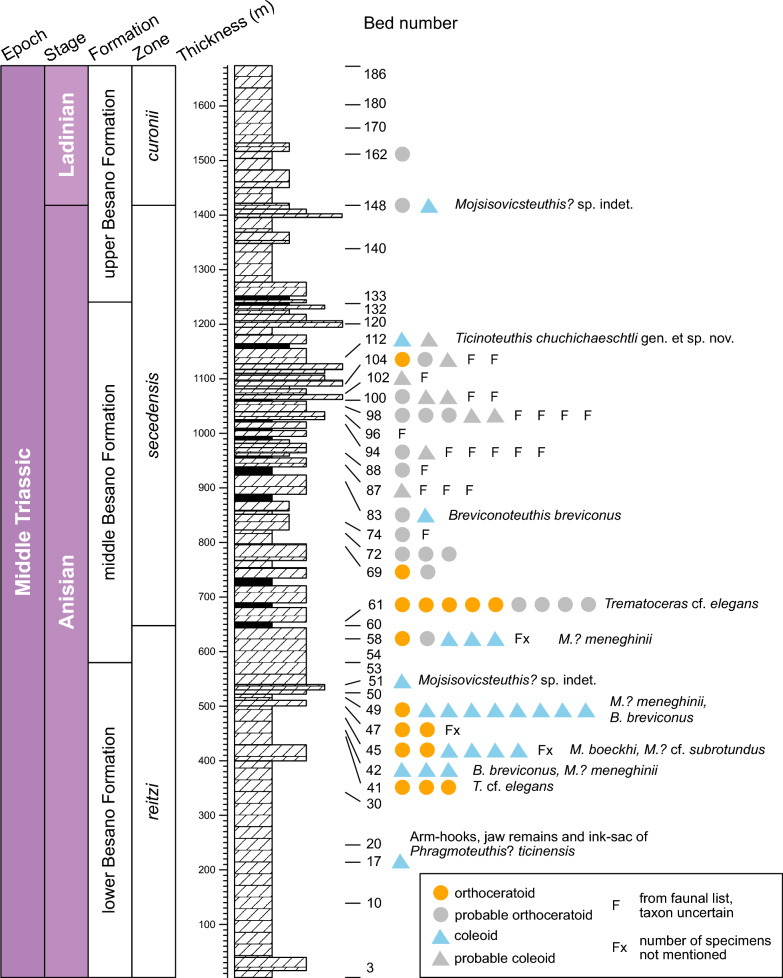
Stratigraphic distribution of orthoconic cephalopods in the Besano Formation at Point 902 and corresponding strata at Monte San Giorgio. Each symbol represents one specimen. Stratigraphy modified after Röhl et al. (2001) and Pieroni (2022)

**Fig. 2:**
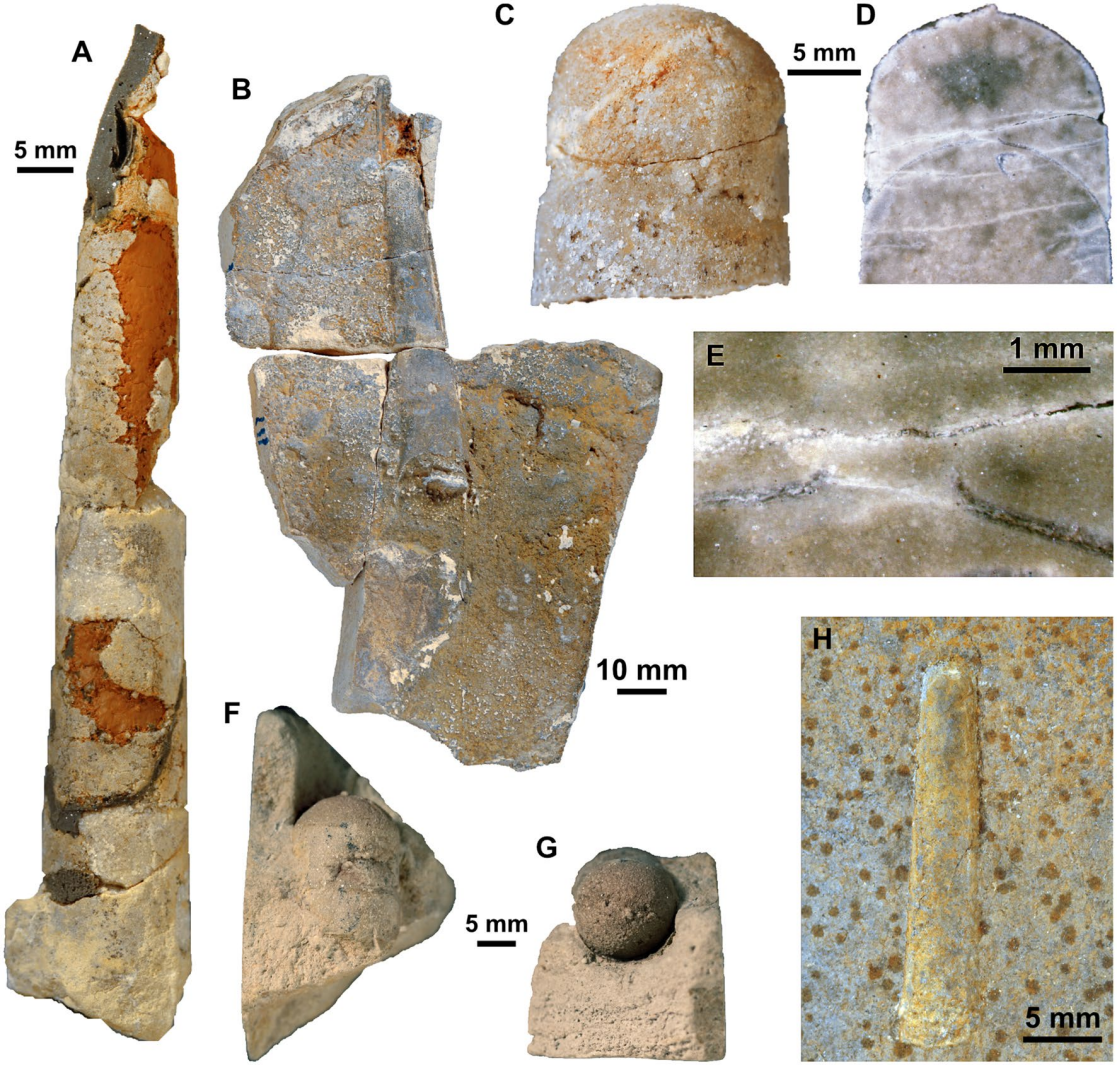
Orthoceratoids from the Besano Formation (Middle Triassic) of the Monte San Giorgio, Ticino, Switzerland. **A–H:** *Trematoceras* cf. *elegans* (Münster, 1841). **A** PIMUZ 39056, partially fixed with silicon, bed 41. **B** PIMUZ 39487, bed 87. **C-E** PIMUZ 39586, sectioned specimen, bed 45. **F–G** PIMUZ 39029, bed 61. **H** PIMUZ 39064, bed 162

The evaluation of the collection in Zurich and the faunal list of Point 902 (provided by H. Furrer, Zurich) revealed that orthoconic cephalopods occur regularly in the Besano Formation within the interval between horizons 40–110 (Fig. 1), though they are always subordinate faunal elements. They usually occur in dolomite beds and are often associated with much more numerous ammonoids and other molluscs. The maximum number of occurrences is in bed 61, where nine orthocones are present in the PIMUZ collection, likely all representing orthoceratoids due to their circular cross sections and small apical angles (Fig. 2). Another peak occurs in bed 45, where four coleoids, described by Rieber (1973) as *Mojsisovicsteuthis boeckhi* and *M*.? cf. *subrotundus*, and two orthoceratoids were found. The breviconic phragmoteuthid *Breviconoteuthis breviconus* has been described from 21 specimens from beds 41, 42, 45, 47, 49 and 83 by Rieber (1973). Another concentration of orthocones occurs between beds 94–104, though most are preserved only as external moulds and provide limited morphological information. The majority of these specimens appear to belong to orthoceratoids, although probable coleoids are also present. The faunal list from Point 902 does not differentiate between orthoceratoids and coleoids but added further occurrences. Although it is not clear, whether the list refers to specimens already present in the collection, several occurrences exceeded the number of specimens present in the collection, e.g., five specimens in bed 94 and one specimen in bed 96. In beds 45, 47 and 58, the number of specimens was not indicated, likely referring to a generally high abundance of specimens in these particular horizons. This agrees with the moderately high number of specimens present from these beds and data from Rieber (1973).

### Diversity

In total, the PIMUZ collection contains 72 specimens of coleoids and orthoceratoids from the Besano Formation, 36 of them probably representing orthoceratoids, 21 phragmoteuthids and 15 likely coleoids of uncertain affinity (including *Ticinoteuthis chuchichaeschtli* gen. et sp. nov., *Mojsisovicsteuthis* and *Phragmoteuthis*? *ticinensis*). Thus, both orthoceratoids and coleoids are much more common in the Besano Formation than nautilids (Pieroni, 2022), but significantly less common than ammonoids (Rieber, 1973). Coleoids have been much better sampled in the past, as our sample includes orthoceratoids from at least 13 additional beds when compared to Rieber (1973), who reported *Michelinoceras campanile* from five beds. At the same time, the only additional coleoids that we recovered likely belong to a new species, which is easily confused with orthoceratoids due to its slender apical angle (see below). Note that these numbers do not necessarily reflect the natural abundance of the taxa, as they were not collected systematically, and it is possible that for example external moulds were often not sampled.

**Fig. 3:**
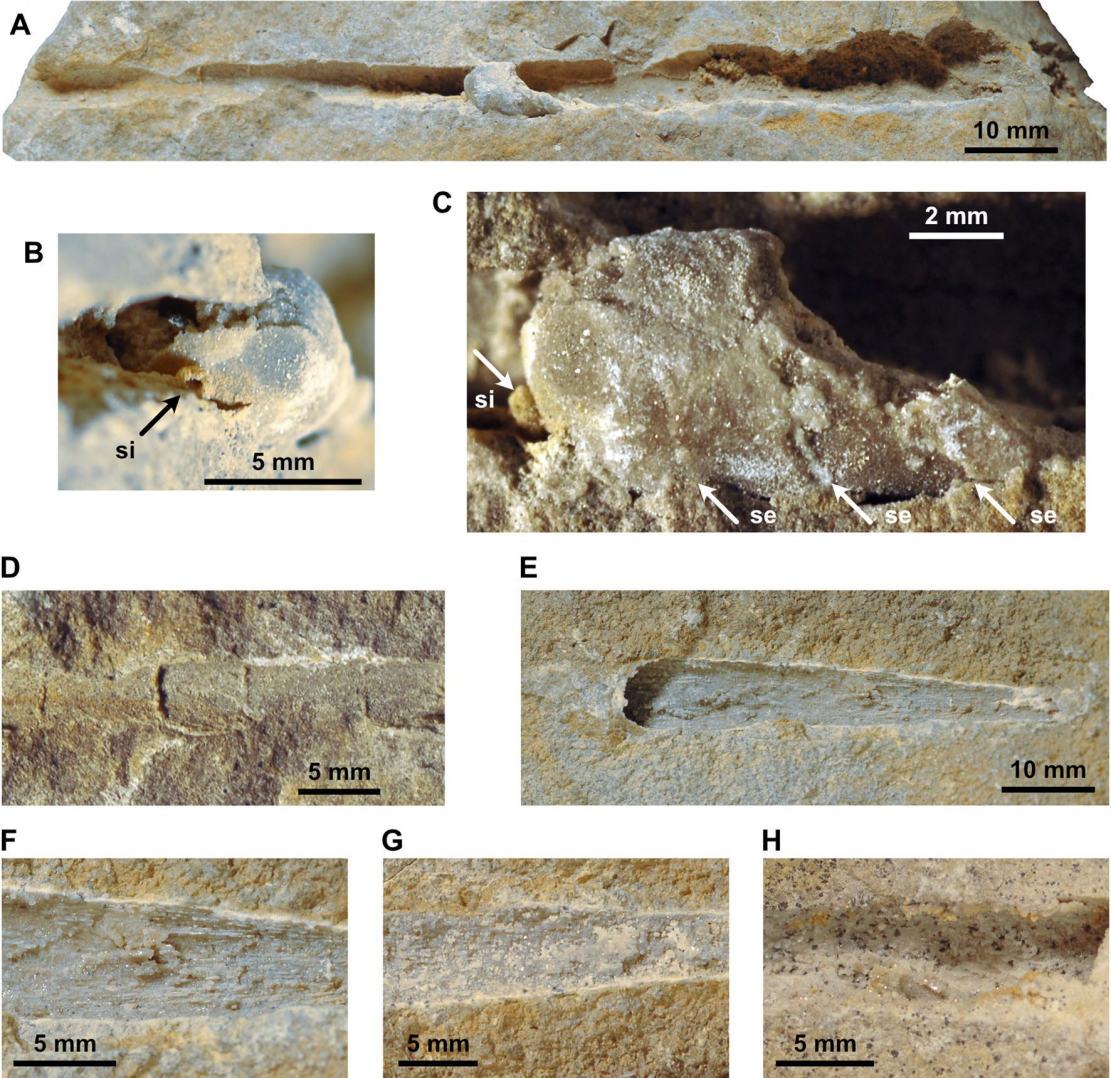
*Ticinoteuthis chuchichaeschtli* gen. et sp. nov. from the Besano Formation (Middle Triassic) of the Monte San Giorgio, Ticino, Switzerland and a co-occurring orthoceratoid for taphonomic comparison. **A–D** PIMUZ 39491, holotype, bed 112 **A** Lateral view. **B** Adapical view of preserved phragmocone, exposing the ventral position of the siphuncle. **C** Lateral view of preserved phragmocone, exposing faint traces of inclined septa. **D** Lateral view of adapical external mould, exposing potential traces of septa. **E–G** PIMUZ 39493, *T. chuchichaeschtli* gen. et sp. nov., bed 94. **E** Lateral view. **F** Enlarged view of longitudinal ribs. **G** Lateral view of counterpart. **H** PIMUZ 39060, bed 94, likely orthoceratoid with similar longitudinal ribs. Note the irregularity in these structures, suggesting that these represent a taphonomic artefact. Abbreviations: si = siphuncle, se = septa

The coleoids mentioned by Rieber (1973) from the Besano Formation are easily distinguishable from the orthoceratoids already by their large apical angles. However, between bed 87 and 112 (i.e. within the *secedensis* zone), there are some orthocones that show a distinct elliptical cross section in combination with on average slightly higher apical angles than the orthoceratoids (Fig. 3). The phragmocone is partially preserved only in a single specimen that possesses a marginal siphuncle (Fig. 3B). The position of the siphuncle reveals that the cross section is dorsoventrally depressed rather than laterally compressed (wider than high). This and further morphological details provide evidence for another, hitherto unrecognised coleoid taxon in the Besano Formation and is here established as *Ticinoteuthis chuchichaeschtli* gen. et sp. nov. (see systematic section). Previously known taxa include *Breviconoteuthis breviconus*, *Mojsisovicsteuthis boeckhi*, *M.? meneghini*, *M.?* cf. *subrotundus* and several other specimens tentatively attributed to *Mojsisovicsteuthis* by Rieber (1973). *Phragmoteuthis? ticinense* is slightly older (bed 17, lower Besano Formation), but is only known from the holotype PIMUZ 3784 preserving arm hooks, jaw remains, cephalic cartilage and ink sac (Rieber, 1970). As the shell of the latter is unknown, it is possibly conspecific with another coleoid species from higher up in the Besano Formation. The preceding coleoid taxa are here only included in the stratigraphic part because they have already been described in detail by Rieber (1973, 1974). The newly reported coleoid material is most similar to the enigmatic *Mojsisovicsteuthis*, which has been reported from slightly older parts of the Besano Formation, as mentioned above (Rieber, 1973). *Ticinoteuthis* gen. nov. is distinct from *Mojsisovicsteuthis* not only in its cross section, but also in the lower apical angle and the apparently retrochoanitic septal necks. The probable lack of a rostrum in both of these taxa suggests affinities to the Phragmoteuthida; however, these differ in having a much shorter, breviconic conch (Fuchs & Donovan, 2018). Furthermore, some of Rieber’s (1973) specimens tentatively assigned to *Mojsisovicsteuthis* preserve part of the body chamber, which would again indicate aulacoceratid relationships. It is thus possible that the two genera represent an intermediate morphotype between aulacoceratids and phragmoteuthids. In any case, more research is needed to clarify the relationship between these Triassic coleoids (see also Košťák et al., 2023).

### Morphology

**Fig. 4:**
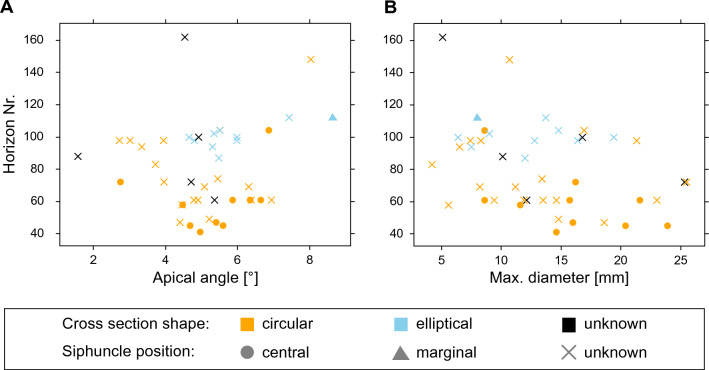
Morphometrics of orthoconic cephalopods from the Besano Formation. Measurements are compared with discrete characters of the shell. Orange circles represent definite and orange crosses likely orthoceratoids, while blue triangles represent definite and blue crosses likely coleoids. Black crosses are indeterminable. **A** Apical angle, calculated from length and diameters of the specimens. **B** Maximum diameter

Morphologically, we cannot discern any obvious trends, partly because there are relatively few specimens per bed (on average 2.7 specimens, see Fig. 1) and their taxonomic identity is not always clear. However, it appears that the apical angle falls within a relatively narrow range between about 3 and 7° (mean = 4.9°, *n* = 34) for the orthoceratoids and slightly higher for the newly reported coleoids, between approximately 5 and 9° (mean = 5.9°, *n* = 10) (Fig. 4A). Note that these measurements exclude specimens of *Mojsisovicsteuthis*, which have a distinctly larger apical angle between 12–25° and *Breviconoteuthis* with apical angles above 30° that is decreasing towards the anterior (Rieber, 1973). There is a slightly higher prevalence of lower apical angles in orthoceratoids around bed 100 but it is still within the range of the lower beds. In terms of size, we can also observe no major differences between the beds, the mean diameter at the apertural end was at 14.0 mm and did not exceed 25.5 mm in the orthoceratoids (Fig. 4B). The coleoids were slightly smaller, with a mean diameter of 12.0 mm and a maximum diameter of 19.4 mm. The only coleoid with preserved phragmocone was even smaller, with an apertural diameter of only 8 mm. This contrasts with Rieber’s (1973) *Mojsisovicsteuthis* specimens that have diameters of up to 45.8 mm. Even the easily distinguishable breviconic *Breviconoteuthis* specimens are usually at the upper end of the diameter size range of our orthocones, although they are shorter due to the high apical angles. Other morphological trends are not obvious due to the number of specimens and their state of preservation. However, it is notable that none of the observable characters of the orthoceratoids seem to deviate from those seen in the type species of *Trematoceras*, i.e. a smooth shell surface, short septal necks and moderately spaced septa (about 0.5–1.5 of conch diameter). In conclusion, we cannot find evidence for any temporal patterns in morphology or body size, suggesting that there was only a single morphologically stable orthoceratoid species present. The coleoids show a more specific distribution, i.e. the large and wide-angled *Breviconoteuthis* and *Mojsisovicsteuthis* mainly in the upper part of the lower Besano Formation (around bed 45), and the small and slender *Ticinoteuthis chuchichaeschtli* gen. et sp. nov. in the upper part of the middle Besano Formation (around bed 100).

### Preservation

External moulds were usually relatively long, suggesting that the shells were not transported over a long distance, as orthoconic conchs easily broke into smaller pieces. Internal moulds were usually shorter, but this may be due to breakage during collection, weathering, or partial dissolution during transport and diagenesis. Shell surface was usually not preserved and there is no evidence for elaborate shell ornamentation. However, longitudinal sectioning revealed that the septa and the septal necks were preserved in some specimens (Fig. 2D, E), although the connecting rings are not preserved. As already mentioned by Rieber (1973), there is no evidence for rostra or parts of rostra in the Besano Formation, as would be expected for aulacoceratids (Jeletzky, 1966; Mariotti et al., 2021). Even if dissolved during diagenesis, their imprints would be expected to be preserved. External moulds of aulacoceratid rostra would be easily distinguishable from phragmocones, as they are usually not as regularly conical (e.g., fusiform, adorally constricted or with blunt apex) and contain numerous longitudinal ribs (Mariotti et al., 2021). Even the Xiphoteuthidoidea, which generally have a smooth rostrum surface, are commonly characterised by lateral grooves or median depressions (Mariotti et al., 2021). One specimen that is likely attributable to *Ticinoteuthis chuchichaeschtli* gen. et sp. nov., PIMUZ 39493, consists of an external mould that bears discontinuous longitudinal ridges that have some resemblance to the imprints of an aulacoceratid rostrum (Fig. 3F). However, they are already more irregular on the counterpart (Fig. 3G). In addition, another specimen that likely represents an orthoceratoid phragmocone (based on traces of septa on a partial internal mould) bears similar ridges that are more diffuse in their orientation, thus rather representing taphonomic artefacts (Fig. 3H).

### Evaluation of taxonomic significance of orthoceratoid morphological characters

The material from Monte San Giorgio cannot fill the significant knowledge gap that still exists for Triassic orthoceratoids. After reviewing the older literature, however, we can now clarify some previous misunderstandings and provide a more modern, albeit still somewhat tentative classification of Triassic orthoceratoids. The fact that orthoceratoids are exceedingly rare in the earliest Triassic, but widespread during the Middle to early Late Triassic combined with their relatively narrow range of morphological variation suggests that they likely form a monophyletic group. Below, we consider different parts of the shell to evaluate their taxonomic relevance, both for the taxonomic position within the Orthoceratoidea and potentially useful characters to distinguish between different Triassic species.

#### Shell surface

In general, Triassic orthoceratoids do not exhibit (or do not preserve) conspicuous conch ornamentation. Nevertheless, some variation has been observed and used to distinguish between taxa. Kutassy (1927) used the shell surface to subdivide Triassic orthoceratoids into three groups, based on previous work by Barrande (1865–1877), Waagen (1879) and Mojsisovics (1873). The first group, “Orthocerata laevia” was designated as having smooth shells or very fine transverse growth lines, containing the species *Orthoceras dubium* Hauer, 1847, *O*. *shastense* Hyatt & Smith, 1905 and *O. triadicum* Mojsisovics, 1873. The second group, “Orthocerata striata” contained the largest number of species in his concept. This group was further subdivided into three subgroups, which were characterised either by transverse striae, longitudinal striae or both, creating a reticulate pattern in the latter case. The transversely striated species represented the largest subgroup, comprising *O*. *elegans* Münster, 1841, *O. subellipticum* d’Orbigny, 1850, *O. politum* Klipstein, 1843, *O. styriacum* Mojsisovics, 1873, *O. celticum* Mojsisovics, 1873, *O. sandlingense* Mojsisovics, 1873, *O. billimiense* Gemmellaro, 1904, *O. subtiliseptatum* Gemmellaro, 1904 and *O. lytosiphon* Gemmellaro, 1904. Meanwhile, he only assigned *O. salinarium* Hauer, 1846 to the longitudinally striated subgroup, and additionally a specimen described as *O.* cfr. *pulchellum* Hauer, 1849 in Gemmellaro, (1904), which Kutassy (1927) considered to be distinct. The last subgroup with reticulate surface ornamentation consisted of the species *O. austriacum* Mojsisovics, 1873 and *O. pulchellum* Hauer, 1849. Lastly, as a third major group of Triassic orthoceratoids, Kutassy (1927) designated the “Orthocerata nodosa”, in which he included his new species *O. nodosum* Kutassy, 1927 and questionably *O. lateseptatum* Hauer, 1846. According to him, these species are characterised by irregular nodes or tubercles on the shell surface, but otherwise exhibit either smooth or transversely striated shells (Kutassy, 1927).

Since most of the above-mentioned species have only been published as drawings, it is difficult to judge the relevance of the shell structure for taxonomic purposes. The “Orthocerata nodosa” group appears questionable at best, and the irregular distribution of the tubercles across the shell together with apparently different types of ornamentation makes it likely that it represents a taphonomic artefact. Similarly, the impact of preservation on shell sculpture is not clear, and perhaps several members of the “Orthocerata laevia” group have just poorly preserved or recrystallised shells. The same may even be true for the various forms of longitudinal or reticulate patterns, which may simply represent cases of well-preserved details. Although it is conceivable that some of this variation in shell sculpture is caused by biological differences, the range of variation is exclusively confined to more or less smooth shells. This becomes particularly obvious when compared to Palaeozoic orthoceratoids, where shell sculpture shows a much greater variation (compare Barskov, 2018; Sweet, 1964). Thus, although shell ornamentation is possibly useful to distinguish between species, the variation does not vary beyond smooth shells or fine growth lines. It thus provides no evidence for a high taxonomic distinctness among Triassic orthoceratoids.

#### Body chamber

In some cases, species have been proposed based on features of the body chamber. For example, Mojsisovics (1869) listed among the characters to distinguish between “*Orthoceras*” *dubium* and “*O.*” *campanile* the length of the body chamber, though neither mentioning intraspecific or ontogenetic variability nor providing threshold values between the two species. In fact, it is not even clear whether he had the absolute body chamber length or merely the proportion of the body chamber in comparison with total length or diameter in mind. Thus, from a modern perspective, the taxonomic value of this character cannot be evaluated without additional data showing its variability. As the length of the body chamber plays an important role in the hydrostatic properties in orthoconic and generally in ectocochleate cephalopods (Peterman et al., 2019), it is conceivable that environmental perturbations or predation could cause plasticity in the length of the body chamber. In modern *Nautilus*, the relative length of the body chamber varies and is directly proportional to the amount of cameral liquid (Collins & Ward, 2010; Collins et al., 1980; Ward et al., 1981). In Triassic ammonoids, it was influenced demonstrably by syn vivo bivalve epibionts (Klug et al., 2004). Furthermore, the chamber formation cycle alone would already cause variation in the length of the body chamber, particularly in species with large septal spacing, if the time shortly before and after the formation of a new septum is compared. In ammonoids, chamber formation was often quite irregular (Siegel et al., 2022; Tajika et al., 2015, 2020), which is usually linked with variations in body chamber length.

Apart from the length of the body chambers, certain modifications of the body chamber have been used to establish species as well. Again “*O.*” *dubium* and in addition “*O.*” *multilabiatum* were described by Hauer (1888) as having constrictions on the internal mould of the body chamber, either only terminally as in the former species, or multiple as in the second species. The problem is that constrictions of the body chamber are a common feature among ectocochleate cephalopods (Klug et al., 2015b) and the absence of these structures may simply indicate an immature specimen. Additionally, body chambers are not even known for most Triassic orthoceratoids preventing species assignments on this basis, thus making it a not particularly useful character. The assessment of the taxonomic value of body chamber constrictions is further diminished by the fact that these constrictions represent an internal thickening of the shell wall and are usually not visible on specimens with preserved shell surface (Hauer, 1888).

The taxonomic value of body chamber features is therefore impeded by uncertain intraspecific and ontogenetic variation, in addition to a general lack of data. Until it is convincingly demonstrated that there are distinct morphotypes of the body chamber, we consider this character as of very limited taxonomic value or at least not a useful character. This also extends to higher taxonomic levels, where the body chamber has never been used to distinguish between orthocerids and pseudorthocerids. As a side note, the body chamber is another source of misidentification in the Triassic: isolated body chambers are usually difficult to assign to coleoids or orthoceratoids, especially if the last septum is missing.

#### Adult size

Although no species have been delimited based on size alone, some authors noted apparent differences between species. For example, Mojsisovics (1882) noted that *“O.” elegans* was smaller than the slightly older *“O.” campanile*. However, Quenstedt (1849) reported that larger specimens also occur in the Cassian Formation, the type horizon of *“O.” elegans*, and according to Leonardi and Polo (1952), the distinction between the two species based on size is virtually impossible, because intermediate specimens including possible juveniles of the larger species do exist. Triassic orthoceratoids appear to be considerably smaller than their Palaeozoic precursors, although size data are poor for most species. The specimens described by Rieber (1973) appear to represent the upper size range with diameters of up to 25 mm. The 16 specimens listed as holotypes and paratypes by Schastlivceva (1988) from different parts of Russia and Kazakhstan do not exceed diameters of 17 mm. The few known Japanese orthoceratoids have diameters below 10 mm (Niko & Ehiro, 2020; Niko et al., 2016) and the fragmentary specimens from Thailand are even smaller (Tongtherm & Nabhitabhata, 2018; Tongtherm et al., 2016). Quenstedt’s (1849) specimens with a diameter of “7/4 inch” (ca. 4–5 cm, depending on which scale Quenstedt was using) from the Cassian Formation of Northern Italy appear to be the largest reported orthoceratoids from the Triassic, although due to the lack of deposited specimens or illustrations in his report, it cannot be excluded that Quenstedt was looking at aulacoceratids. The question arises to which degree sampling influences this size range, since often, large individuals are rarer because of the low survivorship (see discussion in Pohle & Klug, 2018a). This overview shows that there are certainly size differences correlated to stratigraphy and palaeogeography but the poor documentation and more importantly the difficulty in identifying adult specimens render this character a poor species delimiter. Such a definition would also be impractical, as most specimens represent phragmocone fragments that would be impossible to identify under this concept.

#### Conch shape

In contrast to Palaeozoic orthoceratoids, the general conch shape of Triassic members exhibits low variation. Except for the apex, all Triassic orthoceratoids are virtually straight with a constant apical angle that does not appear to change significantly during ontogeny. In general, the apical angle is highly constant across species and does not appear to deviate much from around 5°. The expansion rate is much more variable in Palaeozoic orthoceratoids (compare, e.g., the variation within species of *Orthonybyoceras* from the Ordovician of Estonia, Kröger & Aubrechtová, 2018), but it is particularly low in *Michelinoceras michelini* (Barrande, 1866). Visual comparison of orthoceratid and pseudorthoceratid genera listed in the Treatise (Sweet, 1964) suggests that there may be a tendency towards slightly larger expansion rates in pseudorthoceratids, although we did not test this and there is certainly a considerable overlap. The cross section is almost always circular in Triassic species, with some of the few exceptions being *Orthoceras subellipticum* d’Orbigny, 1850 and *Phatthalungoceras srisuki* Tongtherm & Nabhitabhata, 2018. For both species, taphonomic deformation cannot be excluded, and their original cross section may have been circular as well. *Trematoceras mangishlakense* Schastlivceva, 1981 was described as having a compressed cross section, but the figures indicate that it is only subtle; there may be some taphonomic deformation involved as well. Lastly, *O. styriacum* Mojsisovics, 1873 has an elliptical cross section, but its shell sculpture is so unusual among Triassic orthoceratoids and its internal characters unknown that it cannot be excluded that it actually represents a coleoid.

Therefore, the conch shape of Triassic orthoceratoids is consistently that of a typical orthocone within a very narrow window of variation. Only very few exceptions are known that deviate from this pattern, though all of these possibly resulted from taphonomic processes. The orthoconic shell shape alone does not allow to assign Triassic taxa to any higher group of orthoceratoids. The low variation of these characters also makes distinguishing between species tenuous, but supports the hypothesis that they are closely related.

#### Cameral length

Many differential diagnoses of Triassic species rely to a large degree on septal spacing. To take the Monte San Giorgio specimens of Rieber (1973) as an example, they were assigned to *“Michelinoceras” campanile* (Mojsisovics, 1869) because of the shorter body and phragmocone chambers when compared to *“Michelinoceras” dubium* (Hauer, 1847). However, similar to the length of the body chamber discussed above, there is no study statistically comparing intraspecific or ontogenetic variation in septal spacing in Triassic orthoceratoids. Bizzarini and Gnoli (1991) mentioned that relative phragmocone chamber lengths (RCL; see Pohle et al., 2022 supplementary material) can vary within the same specimen as much as between 0.5 and 1.5, which almost covers the entire range of known cameral lengths in Triassic orthoceratoids. It is known from many other ectocochleate cephalopods that cameral length can be highly variable, even within the same specimen (Siegel et al., 2022; Tajika et al., 2020). This can be related to septal crowding near maturity or simply indicate phases of slower growth (e.g., Tajika & Klug, 2020; Ward, 1985).

However, discounting cameral length entirely is not justified either. A few Triassic orthoceratoids, particularly some Early Triassic forms, are characterised by consistently short chambers. This might correlate with expanded siphuncular segments as in species assigned to *Pseudotemperoceras* or *Phatthalungoceras* (see below). Ideally, rather than reporting a single value for septal spacing, it would be desirable to report ontogenetic trajectories of cameral length, which can then be more reasonably compared.

#### Siphuncle

**Fig. 5:**
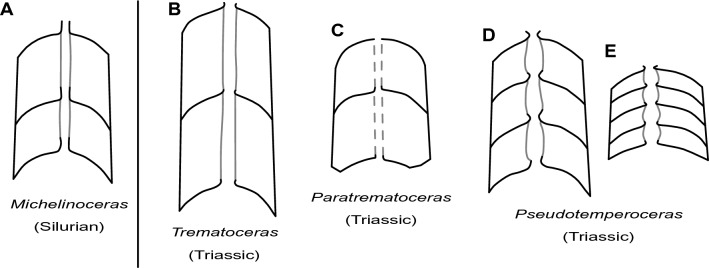
Camera lucida drawings of Triassic orthoceratoid genera (not to scale) in comparison with *Michelinoceras*. *Phattalungoceras* is not shown here, as the only specimen is too poorly preserved to confidently reconstruct its internal structures, although it appears to resemble *Pseudotemperoceras*. **A** *Michelinoceras michelini*, after Barrande (1870; pl. 381; fig. 14); **B** *Trematoceras elegans*, after Schindewolf (1933; fig. 5); **C** *Paratrematoceras shevyrevi*, after Schastlivceva (1988 pl. 1, fig. 3); **D** *Pseudotemperoceras nyalamense*, after Chen (1981; text-fig. 2); **E** *Pseudotemperoceras pulchrum*, after Schastlivceva (1988; pl. 1, fig. 5c)

In comparison with external shell characters, the morphology of the siphuncle is more variable. There are two main morphotypes, one with short orthochoanitic septal necks and tubular siphuncular segments (Fig. 5B, C) and another one with slightly longer, suborthochoanitic necks and expanded segments (Fig. 5D, E). The first morphotype appears to be more common and is characteristic of *Trematoceras*, which has a relatively wide siphuncle (Fig. 5B) and *Paratrematoceras*, which has a narrower siphuncle and more sharply bent septal necks (Fig. 5C). However, the amount of variation in this character and the potential influence of the position of the sectioning plane have not been investigated, so it is possible that these two genera are in fact synonymous. For now, the difference seems large enough to warrant their separation. The second morphotype has only been reported from few specimens, two of which have been assigned to the genera *Pseudotemperoceras* and *Phatthalungoceras*, respectively.

#### Cameral deposits

Some authors attempted to distinguish *Trematoceras* from other Triassic orthoceratoids by the presence of its peculiar cameral deposits (e.g., Schastlivceva, 1988; Silberling & Nichols, 1982). Accordingly, specimens without cameral deposits would be assigned to *Michelinoceras*. However, this approach ignores the heterochronous growth of cameral deposits, i.e. two fragments of the same size may represent different ontogenetic stages and thus either lack or bear cameral deposits (Flower, 1964). Since the apicalmost part of the conch is usually missing, fragments lacking cameral deposits may come from specimens where cameral deposits are restricted to more apical regions. Furthermore, the preservation potential of cameral deposits has not been studied and therefore, absence does not necessarily imply primary absence but may also be caused by taphonomic processes. Moreover, many species of Triassic orthoceratoids have only been described based on their external characters. Thus, for all these species, assignment to either genus would be impossible without restudying the type or additional material. In conclusion, cameral deposits cannot serve as the sole criterion to separate the two genera, as only *Trematoceras* could be identified with confidence.

The structure of the cameral deposits has only rarely been investigated in Triassic and other orthoceratoids. Dauphin (1989) performed geochemical analyses on Late Triassic “*Michelinoceras*”, confirming that they differ in composition from both the sediment and the primary shell. The most recent and most detailed photographs of the three-dimensional structure of the cameral deposits of *Trematoceras* were published by Bizzarini and Gnoli (1991), which confirmed the unique radial pattern first reported by Barrande (1865–1877). This very regular symmetric pattern and the repetition of cameral deposits in subsequent chambers makes it unlikely that cameral deposits are generally a post-mortem product involving bacteria as claimed by Mutvei (2018). Similarly regular though considerably different patterns occur in Devonian orthoceratoids, further supporting a biogenic origin of cameral deposits in general (Pohle & Klug, 2018b).There is little known about the variation of cameral deposits between different species of Triassic orthoceratoids. As the characteristic radial shape is the only one that has been reported, it is so far unknown if other types exist. In several Triassic orthoceratoid species, cameral deposits are well visible in longitudinal sections (e.g., Niko & Ehiro, 2020; Niko et al., 2016; Schastlivceva, 1981, 1986, 1988; Schindewolf, 1933), but they have not been investigated in 3D. Cameral deposits are generally poorly understood and more research on their formation, ontogeny, composition and potential taphonomic biases is needed before they can be used as a character to distinguish different taxa (e.g., Blind, 1991; Dauphin, 1989; Fischer & Teichert, 1969; Flower, 1955; Mutvei, 2018; Pohle & Klug, 2018b; Seuss et al., 2012).

#### Endosiphuncular deposits

Organic deposits within the siphuncle are common and diverse in many groups of Palaeozoic cephalopods (Teichert, 1964). The shape of these endosiphuncular deposits has been used to differentiate between some of these groups and plays a particularly important role in orthoceratoids (Flower, 1939; Hook & Flower, 1976; King & Evans, 2019; Teichert, 1964). For example, the Pseudorthocerida have “parietal” deposits that are elongated in adapertural direction, while the “annular” deposits of the Orthocerida grow symmetrically from the septal necks, often confined to the vicinity of the septal necks (Flower, 1939; Teichert, 1964). The endosiphuncular deposits of Triassic orthoceratoids have rarely been mentioned, although they appear to be mostly of the parietal type (Jeletzky & Zapfe, 1967; Niko & Ehiro, 2020; Schastlivceva, 1986, 1988), although Schastlivceva (1986, 1988) described the endosiphuncular deposits in *Pseudotemperoceras* as annular, thus assigning it to the Geisonoceratidae of the Orthocerida. However, as with the cameral deposits, there is little known about the ontogeny of the endosiphuncular deposits, and therefore, they are currently of limited use at a low taxonomic level.

#### Embryonic shell

**Fig. 6:**
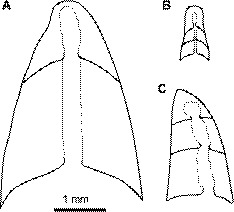
Comparison of embryonic shells of selected orthoceratoids. **A** *Trematoceras* cf. *politum* (= *T. elegans*), Late Triassic, Carnian, after Schindewolf (1933; fig. 5). **B** *Michelinoceras michelini*, late Silurian, Ludlow, after Ristedt (1968; fig. 3.1b); **C** *Pseudorthoceras knoxense*, Carboniferous, Serpukhovian, after Kröger & Mapes (Kröger & Mapes, 2004; fig. 5.9)

The embryonic shell of *Trematoceras* (Fig. 6A) including the type species *T. elegans* (Münster, 1841) is comparatively well known, and a number of apices have been described (Barrande 1865–1877; Hyatt, 1884; Schindewolf, 1933; Leonardi & Polo, 1952; Erben & Flajs, 1975; Bizzarini & Gnoli, 1991). All of these apices are conical and have a prominent protrusion at the tip, which corresponds to the cicatrix according to the definition of Erben and Flajs (1975). The apex is slightly curved, leading to a subcentrally to eccentrically positioned cicatrix. The variation is evident in material from the Cassian formation, where *Orthoceras politum* var. *acumitatum* displays a very strongly eccentric apex (Leonardi & Polo, 1952; pl. 2; fig. 56), while it is only slightly subcentral in *Orthoceras politum* (Leonardi & Polo, 1952; pl. 2; fig. 44, 46, 48, 50). The cicatrix has been suggested to be dorsal in *Trematoceras* (Kröger & Mapes, 2004), although interpreting the orientation of the animal is difficult. If this interpretation is correct, then the apex of *Trematoceras* is exogastrically curved, as in many other pseudorthocerids. Internally, the apex consists of a short initial chamber and an elongated, slightly expanded caecum that is close, but not attached to the shell wall. These characteristics are known from two apparently distinct species, which show a variably prominent size difference, but otherwise agree in the internal structure of the initial chamber (Schindewolf, 1933; fig. 5–6). There is some size variation in the known apices, but they appear to fall within a range of about 1–2 mm diameter at the first septum. In summary, although there is variation in the embryonic shell of *Trematoceras*, these differences are relatively minor and probably do not account for more than differences between species. Unfortunately, all previously documented apices apparently originate from the Cassian Formation in northern Italy—although neither Hyatt (1884) nor Erben and Flajs (1975) stated the origin of their apices. Thus, further embryonic shells from other areas and stratigraphic positions—particularly of *Paratrematoceras* or *Pseudotemperoceras*—would be invaluable to further elucidate taxonomic relationships between Triassic and earlier orthoceratoids.

Regardless of the limited taxonomic, geographic and stratigraphic coverage of the Triassic orthoceratoid apices, they differ in several important aspects from those of *Michelinoceras* (Fig. 6C). The embryonic shell of the type species *Michelinoceras michelini* Barrande, 1866 from the late Silurian of Bohemia was described in detail by Ristedt (1968). It can be distinguished from those of *Trematoceras* not only by its dimensions, which reaches only about 0.4 mm in maximum diameter, but also in the subspherical shape of the apex without cicatrix. Additionally, the initial chamber is longer than the subsequent chambers and it is distinctly set off by a higher expansion rate at the first septum (Ristedt, 1968). Therefore, the apex of *Michelinoceras* is much more similar to those of other typical orthocerids (compare Aubrechtová et al., 2020; Kröger, 2006; Kröger & Isakar, 2006; Kröger & Pohle, 2021).

In contrast, the embryonic shell of *Trematoceras* resembles those of pseudorthocerids. The apex of *Pseudorthoceras* Girty, 1911 (Fig. 6B) and particularly of the type species *Pseudorthoceras knoxense* (McChesney, 1860) is probably the best studied among pseudorthocerids and in orthoceratoids in general (Blind, 1988; Kröger & Mapes, 2004; Miller et al., 1933; Ristedt, 1971). It consists of a bluntly conical, exogastric embryonic shell with cicatrix. The initial chamber is shorter than the subsequent chambers and there is no indication of a growth change at the first septum. In addition, the embryonic shell of *Pseudorthoceras* agrees with that of *Trematoceras* in the orthochoanitic septal necks at the first septum, which change to suborthochoanitic in subsequent chambers. Lastly, the diameter of the embryonic shell of *Pseudorthoceras* is closer to *Trematoceras* than to *Michelinoceras*. The size of the embryonic shell is particularly remarkable when considering that most adult specimens do not appear to exceed diameters of 2–3 cm.

## Discussion

### Coleoids in the Besano Formation

It is interesting to note that in comparison with the orthoceratoids, coleoids are relatively diverse in the Besano Formation, despite their low abundance, with six species reported by Rieber (1970, 1973, 1974) and this study. This pattern somewhat resembles the pattern of the nautilids, which are even rarer in the Besano Formation (Pieroni, 2022). Another interesting pattern is that rostrum-bearing coleoids such as aulacoceratids are apparently completely absent. It is possible that this pattern is caused by taphonomic processes; however, one would expect them to be preserved since recrystalised shell remains are not uncommon (Rieber, 1973). The coleoids described by Rieber (1970, 1973, 1974) and in this study show that Triassic coleoids need more research, because even though their global diversity seems to be lower than in later Mesozoic coleoids (e.g., Fuchs, 2020, 2023), they show considerable variability. *Breviconoteuthis* is a member of the Phragmoteuthida, which are considered to possibly comprise putatitve stem-octobrachians and therefore are of high relevance in deciphering the early evolution of the Coleoidea (Fuchs & Donovan, 2018). *Mojsisovicsteuthis* has remained enigmatic since its description by Jeletzky (1966) and does neither fit within the Phragmoteuthida nor in the Aulacoceratida due to the presence of a body chamber and apparent lack of a rostrum proper. Possibly, it can be connected to some of the Palaeozoic coleoid groups, but knowledge on these taxa is similarly patchy (Fuchs, 2021). *Ticinoteuthis chuchichaeschtli* gen. et sp. nov. is probably related to *Mojsisovicsteuthis* and thus represents a welcome addition to the knowledge about this type of coleoid. The two genera may represent an intermediate step between rostrum-bearing aulacoceratids and the proostracum-bearing phragmoteuthids through the loss of the rostrum and transformation of the body chamber into a proostracum. However, more and better preserved material is required to better understand their phylogenetic affinities. It also shows that orthoconic phragmocones potentially provide further clues to early coleoid evolution that are easily overlooked by focussing on rostra and proostraca.

### Taxonomic position of Triassic orthoceratoids

**Fig. 7:**
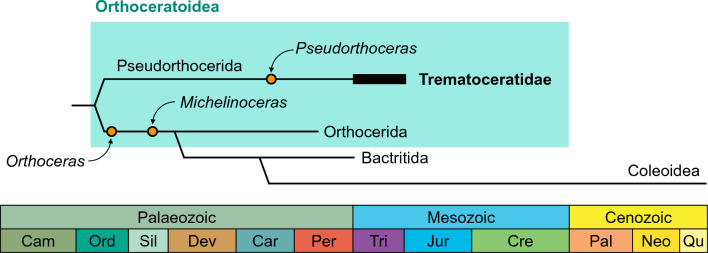
Simplified time-scaled phylogeny demonstrating possible relationships of the Trematoceratidae to other orthoceratoids and coleoids. Note that *Orthoceras* and *Michelinoceras* are more closely related to coleoids than they are to trematoceratids under this concept. Several groups are omitted here for clarity (e.g., Nautilida, Ammonoida). The questionable *Zhuravlevia* is here excluded

There are two main takeaways gathered from the consideration of different parts of the shell. First, Triassic orthoceratoids are generally morphologically uniform, both externally and internally. Together with their scarcity in the earliest Triassic and subsequent recovery during the Middle Triassic, it is probable that only a single lineage survived the end-Permian mass extinction (dead clade walking; Jablonski, 2001), rather than three independent families convergently evolving nearly identical conch morphologies as suggested by Schastlivceva (1988). Thus, we propose that all Triassic orthoceratoids are more closely related to each other than to any other taxon, thus belonging to a single family, the Trematoceratidae (Fig. 7). Unfortunately, intraspecific and ontogenetic variability are largely unknown for most Triassic species, leading likely to an oversplitting of species. Attempts to revise the taxonomy of the Trematoceratidae should thus involve reconstructing ontogenetic trajectories of conch parameters. The most variable and thus most promising characters for the low-level taxonomy appear to be the shape of the siphuncle and the embryonic shell. Further studies on the structure, growth and taphonomy of endosiphuncular and cameral deposits may also help in delimiting species in the future. The second takeaway is that *Trematoceras* and other Triassic orthoceratoids show many characteristics that are typical for pseudorthocerids rather than orthocerids. The only possible exception to this pattern is the Early Triassic *Pseudotemperoceras*, which has been described with annular deposits that may indicate a geisonoceratid affinity (Schastlivceva, 1988), although more research is needed that would confirm their taxonomic separation from pseudorthocerids. Alternatively, the “annular” may simply represent an earlier ontogenetic stage of the endosiphuncular deposits or they may be more restricted to the septal necks in these species. The latter pattern is also common in Ordovician orthoceratoids (Kröger, 2004). Otherwise, the association of the Trematoceratidae with the Pseudorthocerida is well justified. Even though embryonic shells are only known from a limited geographic and stratigraphic range, the overall similarity between trematoceratids implies that similar embryonic shells would also be expected for other species. In any case, unless a clear case for true Orthocerida in the Triassic can be made, our taxonomic framework represents the hypothesis that best explains the available evidence with the least amount of (poorly supported) assumptions. This preliminary revised taxonomy is shown in Table 2, listing only species that are sufficiently known from internal characters to be accepted.

**Table 2:** Updated systematic classification of Triassic orthoceratoids (see also Schastlivceva, 1988; Zakharov, 1996)

Class **Cephalopoda** Cuvier, 1797
Subclass **Orthoceratoidea** Teichert, 1967
Order **Pseudorthocerida** Flower & Caster, 1935
Family **Trematoceratidae** Zakharov, 1996
Genus ***Trematoceras*** Eichwald, 1851
*Trematoceras elegans* (Münster, 1841)
*Trematoceras boreale* Schastlivceva, 1986
*Trematoceras clarum* Schastlivceva, 1986
*Trematoceras hikichii* Niko et al., 2016
*Trematoceras insperatum* Schastlivceva, 1988
*Trematoceras lytosiphon* (Gemmellaro, 1904) comb. nov
*Trematoceras mangishlakense* Schastlivceva, 1981
*Trematoceras solidum* Schastlivceva, 1988
*Trematoceras subcampanile* (Kiparisova, 1954)
*Trematoceras vulgare* Schastlivceva, 1981
*Trematoceras watanabei* Niko & Ehiro, 2020
Genus ***Paratrematoceras*** Schastlivceva, 1981
*Paratrematoceras shevyrevi* Schastlivceva, 1981
*Paratrematoceras ornatum* Schastlivceva, 1981
*Paratrematoceras salinarium* (Hauer, 1846) comb. nov.
Genus ***Pseudotemperoceras*** Schastlivceva, 1986
*Pseudotemperoceras pulchrum* Schastlivceva, 1986
*Pseudotemperoceras nyalamense* (Chen, 1981) comb. nov.
Genus ***Phatthalungoceras*** Tongtherm & Nabhitabhata, 2018
*Phatthalungoceras srisuki* Tongtherm & Nabhitabhata, 2018
Genus ***Zhuravlevia*** Doguzhaeva, 1994
*Zhuravlevia insperata* Doguzhaeva, 1994

Only species with sufficiently known internal characters are listed here. All other species are currently too poorly known to allow for generic assignment and may represent synonyms of other species

### Palaeoecology of Triassic orthoceratoids

Considering the absence of soft tissue preservation, interpreting the ecological role of trematoceratids is challenging and remains speculative to some extent. However, the detailed excavations at Monte San Giorgio can at least provide some indirect evidence by revealing distinct distribution patterns. As with their Palaeozoic counterparts, the distribution of trematoceratids appears to be highly facies-dependent, i.e. they are almost entirely restricted to dolomite beds in the middle part of the Besano Formation that represent a deeper, intraplatform basin (Röhl et al., 2001). Their common association with dasycladacean algae, gastropods and bivalves of the genus *Daonella* indicates that they inhabited the photic zone. Ammonoids and coleoids usually occur within the same horizons, though it is difficult to discern distinct taxon-specific patterns due to the general abundance of ammonoids and scarcity of other cephalopods. Coleoids appear to be more numerous in the lower Besano Formation (below bed 53), while trematoceratids appear to be more common above this level. However, as it is not always straightforward to distinguish between the two groups, it is possible that these observations bear no significance. Palaeozoic orthoceratoids frequently form mass occurrences (e.g., Bogolepova & Holland, 1995; Hewitt & Watkins, 1980; Kröger & Pohle, 2021; Pohle & Klug, 2018a), suggesting that they may have formed moderate to large shoals, at least during mating season as in many modern squid. Occurrences of Triassic orthoceratoids are much more isolated and thus, they had perhaps a more solitary lifestyle. This would also be supported by their comparatively large embryonic conchs, suggesting lower reproductive rates (high survivorships, k-strategy) in contrast to the presumably more prevalent r-strategy in Palaeozoic orthoceratoids (Laptikhovsky et al., 2018). Even if the absolute size of the embryonic shell may seem small, it is very large when compared to the size of the adult animal, which is not known to exceed a couple of centimetres in diameter. The actual size at hatching is difficult to estimate, but it is probable that it occurred somewhere around the point where the initially high expansion rate stabilised, i.e. approximately 3–4 mm. It is also notable that the initial chambers of the k-strategist *Nautilus* form at similar whorl diameters (Tajika et al., 2015), even if the embryonic shell itself is much larger (Arnold, 1987; Boletzky, 1988).

### Palaeobiogeography and stratigraphic distribution of Triassic orthoceratoids

**Fig. 8:**
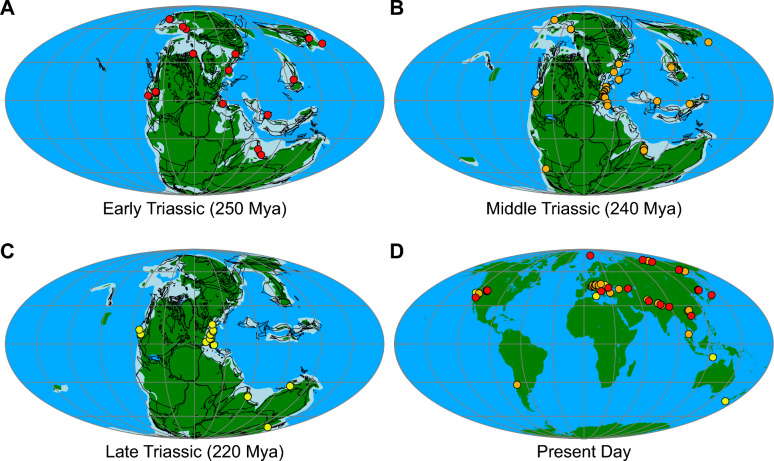
Paleobiogeographic distribution of Triassic orthoceratoids. Palaeogeographic reconstruction produced with GPlates (Müller et al., 2018), with data from Cao et al. (2017) and Müller et al. (2019). Fossil localities of Triassic orthoceratoids from the Paleobiodiversity Database (https://paleobiodb.org) with additional data from the literature. **A** Early Triassic; **B** Middle Triassic; **C** Late Triassic; **D** present day

During the Triassic, trematoceratids had a global distribution (Fig. 8). To our knowledge, orthoceratoids have not yet been reported from Induan deposits, possibly indicating that they underwent an evolutionary bottleneck during that time. *Pseudotemperoceras nyalamense* (Chen, 1981) and the co-occurring “*Michelinoceras* cf. *lytosiphon*” and “?*Michelinoceras* sp. B” from the Early Triassic lower Tulong Formation of Tibet represent perhaps the oldest record of Triassic orthoceratoids (Chen, 1981). The stratigraphy of the Tulong Formation is now better resolved with detailed ammonoid, sedimentary and carbon isotope data (Brühwiler et al., 2009, 2010), though it is unclear from which beds the orthoceratoids were collected. According to Brühwiler et al. (2009), orthoceratoids are common in subunit IVa, which corresponds to the early Spathian and thus the earliest Olenekian. Orthoceratoids are better known from later Olenekian deposits of East Asia (Brühwiler et al., 2009; Kiparisova, 1954; Kummel & Erben, 1968; Niko et al., 2016; Schastlivceva, 1981, 1986, 1988; Shigeta & Nguyen, 2014; Shigeta & Zakharov, 2009), but also from Eastern Europe (Germani, 1997; Grădinaru et al., 2007) and North America (Brayard et al., 2019). In some Olenekian localities, orthoceratoids are rather common, although they have usually not been studied in detail (e.g., Brayard et al., 2019; Brühwiler et al., 2009). It thus appears that despite the short hiatus in the earliest Triassic, trematoceratids recovered relatively quickly and already reached a widespread distribution concentrated in the northern hemisphere during the Early Triassic (although this may be caused by sampling biases; see, e.g., Vilhena & Smith, 2013; Close et al., 2020). Their range extended from the northwestern margin of Pangaea over arctic regions up to the western and southern Tethys. It is remarkable that trematoceratids have been found in both low and high palaeolatitudes, suggesting that they adapted to a range of climatic conditions already early in the Triassic. The maximum generic diversity (and thus morphological disparity) within the Trematoceratidae was apparently already reached during the Olenekian, where the three genera *Trematoceras*, *Paratrematoceras* and *Pseudotemperoceras* were already present (Schastlivceva, 1988). Trematoceratids further flourished during the Middle Triassic, where they are particularly well documented within the western Tethyan realm but have been found in many areas worldwide. While trematoceratids are still relatively well-documented in the Carnian of the Alps, they appear to have had a more restricted range during the Late Triassic. Other known occurrences are from the Carnian of equatorial western Pangaea, i.e. modern day California (Hyatt & Smith, 1905) and from the southeastern coast of Pangaea, i.e. New Zealand and Timor (Bülow, 1915; Trechmann, 1918). Norian orthoceratoids have occasionally been reported although they appear to be much rarer. This indicates that the decline of the group started already before the end-Triassic mass extinction event. This is further corroborated by the fact that only a single Rhaetian trematoceratid has been reported, from the Zlambach Marl of Austria (Jeletzky & Zapfe, 1967).

## Conclusions

Triassic fossils previously referred to either “*Orthoceras*” or “*Michelinoceras*” are better placed in *Trematoceras*, *Paratrematoceras* or *Pseudotemperoceras* of the Trematoceratidae. Consideration of all known morphological characters indicates that they more closely resemble pseudorthocerids rather than orthocerids, particularly with reference to their early ontogeny. They are thus included in the Pseudorthocerida. Consequently, the Orthocerida is an exclusively Palaeozoic taxon. Reports of post-Triassic orthoceratoids (Doguzhaeva, 1994; Doguzhaeva et al., 2017) are so rare that they need further confirmation to exclude alternative interpretations such as reworking or affinities to other cephalopod groups such as coleoids. An Eocene orthoceratoid-descendant has already been re-interpreted as coleoid remains by previous authors (Fuchs et al., 2020; but see Doguzhaeva, 2020). If the Late Cretaceous *Zhuravlevia* Doguzhaeva, 1994 is confirmed by future studies, it is likely that it can be assigned to the Trematoceratidae as well, due to its general similarity in conch shape and internal structures.

## Systematic palaeontology

We tentatively consider all orthocones of this collection with elliptical cross section as coleoids for the following reasons:

1. There are no confirmed Triassic orthoceratoids with non-circular cross sections. Although some species such as “*Orthoceras*” *subellipticum* d’Orbigny, 1850 or “*O*.” *styriacum* Mojsisovics, 1873 reportedly have an elliptical cross section, they may be caused by taphonomic deformation, as indicated already by Quenstedt (1849). Alternatively, as the position of the siphuncle is unknown in both of these species, they may even represent phragmocones of coleoids themselves. In any case, even within Palaeozoic orthoceratoids, elliptical cross sections are comparatively rare (Sweet, 1964).
2. The only specimen with an elliptical cross section that preserves the siphuncle has a marginal siphuncle. Although Palaeozoic orthoceratoids with marginal or submarginal siphuncles are known, Triassic representatives have exclusively central siphuncles (Schastlivceva, 1988).
3. The apical angle of the potential coleoid specimens tends to be higher than in the orthoceratoids (although this may be a result of compaction).

Therefore, these specimens fall distinctively outside the known variation of Triassic orthoceratoids, while they easily fit within the comparatively wider range of variation in Triassic coleoids (Jeletzky, 1966). This interpretation comes with the potential caveat that the elliptical cross sections may have been produced by taphonomic compaction. Nevertheless, the fact that they are distributed approximately around the same stratigraphic level, the regularity of the cross section and the similarity in apical angles suggest that they potentially belong to the same or closely related coleoid species. For specimens with circular cross sections, the interpretation is more difficult because cross sections of Triassic coleoids are more diverse (cf. Fuchs & Donovan, 2018; Iba et al., 2012; Mariotti et al., 2021). However, since there is currently no record of a coleoid that combines a very narrow apical angle with a circular cross section, we tentatively interpret these specimens as orthoceratoids. This is corroborated by the fact that where the siphuncle is visible in these specimens, it is located in a central position—again a feature unknown in coleoids. There are no preserved rostra in the Besano Formation which would allow for a further indication of coleoid affinities (see discussion).

While attempting a taxonomic placement of the orthoceratoids, it became obvious that the taxonomy of Triassic orthoceratoids needs extensive revisions. Recent descriptions of Triassic orthoceratoids are commonly either very superficial, using outdated taxonomic names such as “*Orthoceras*” or *“Michelinoceras*” (cf. Schastlivceva, 1988), or very limited in scope, comparing only few regional species without reference to the large number of older taxonomic names (e.g., Niko & Ehiro, 2020; Niko et al., 2016; Tongtherm & Nabhitabhata, 2018). This has led to an inflation in species names, particularly of the genus *Trematoceras*, which currently has about fifty species assigned to it (see below). To facilitate future descriptions or revisions of the group, it is therefore vital to assemble an overview of the current systematic status of Triassic orthoceratoids, which is at present only available in a more than thirty years old monograph exclusively in Russian language (Schastlivceva, 1988). Therefore, we first summarise previous research on Triassic orthoceratoids, put it into a modern taxonomic context and discuss potential phylogenetic implications. In doing so, we list all previously described genera and species known to us and their geographic and stratigraphic distributions. Note that while we consider synonymies between those species as likely, we here make only a small attempt to revise the low-level taxonomy, as a more extensive revision would require first-hand comparisons of original type material. However, it may provide a useful guide for future taxonomic treatments of the group.

We here describe the coleoid and orthoceratoid material from Monte San Giorgio, but also briefly outline further trematoceratid genera, as current diagnoses are missing from the scientific literature in English language.

Class **Cephalopoda** Cuvier, 1797

Subclass **Coleoidea** Bather, 1888

Order and Family uncertain.

Genus ***Ticinoteuthis*** gen. nov.

urn:lsid:zoobank.org:act: F7E76450-CFD4-4BCA-8D4E-7FF4D6D83094.

**Etymology**: Named after the Canton Ticino (Switzerland), where the type locality is situated.

**Type species**: *Ticinoteuthis chuchichaeschtli* gen. et sp. nov.

**Included species**: Only the type species.

**Diagnosis**: Small orthoconic (longiconic) phragmocone with slender expansion rate of about 5–9° and depressed cross section. Septa apparently inclined towards the venter in anterior direction. Siphuncle submarginal, probably with retrochoanitic septal necks. Neither primordial nor proper rostrum are known.

**Remarks: ***Ticinoteuthis* gen. nov. is similar to *Mojsisovicsteuthis* but differs in its depressed rather than compressed cross section, slenderer apical angle and apparently retrochoanitic instead of prochoanitic septal necks. For both *Mojsisovicsteuthis* and *Ticinoteuthis* gen. nov., a rostrum (or telum) is undocumented, which was interpreted by Jeletzky (1966) as primary absence. If this is the case, then they cannot be assigned to the Aulacoceratida, an approach that was already proposed by Mariotti and Pignatti (1992, 1993) and by the Treatise on Invertebrate Palaeontology (Mariotti et al., 2021). The absence of a rostrum may be taken as evidence to classify these taxa within the Phragmoteuthida, but representatives of this order are generally characterised by much larger apical angles and possess a typical three-lobed proostracum (Fuchs & Donovan, 2018). In contrast, Rieber (1973) described body chambers of *Mojsisovicsteuthis*, which is mutually exclusive with any proostracum. For *T. chuchichaeschtli*, it is unclear if a tubular body chamber or a proostracum was present, although the similarity to *Mojsisovicsteuthis* perhaps suggests a tubular body chamber as well. Thus, they do not fit any of the known Mesozoic coleoid orders or even families, implying that besides desperately needed new data, current family- or order-level definitions either need to be adjusted or new names to be established.

It is possible that “*Orthoceras*” *styriacum* Mojsisovics, 1873 from the Carnian of Austria belongs to the same group of species, perhaps even to *Ticinoteuthis*, as it has a similar expansion rate and cross section and further appears to agree with in the inclination of the septa. However, as the position of the siphuncle is unknown in “*O*.” *styriacum*, it cannot be excluded that it represents a crushed trematoceratid.

Aulacoceratids differ in having a thick aragonitic rostrum, but their phragmocones generally expand more rapidly and are (sub-) circular in cross section. The only aulacoceratid with a similarly depressed cross section of the phragmocone is *Miyagiteuthis*, which further differs in having a strictly marginal siphuncle and less inclined septa (Mariotti et al., 2021; Niko & Ehiro, 2018).

**Occurrence:** Ticino, Switzerland; Middle Triassic (Anisian).

***Ticinoteuthis chuchichaeschtli*** gen. et sp. nov.

urn:lsid:zoobank.org:act:67,831,665-256E-476D-8690-5C75A42D2F44.

Figure 3A-G, 10C

**Etymology**: The Swiss German word “Chuchichäschtli” is a well-known shibboleth translating to small kitchen cupboard (standard German = Küchenschränkchen). This is a reference to the specimens being stored in a drawer in Zurich for many years.

**Holotype**: PIMUZ 39491 is the only specimen that shows the siphuncle and a partly preserved phragmocone and therefore selected as holotype.

**Type locality and horizon**: Point 902/Mirigioli, Meride, Ticino; middle Besano Formation, bed 112, *secedensis* zone, Illyrian, Anisian, middle Triassic.

**Material**: Two further specimens are preserved as internal moulds without trace of septa or siphuncle (PIMUZ 39487, 39,489) and seven further specimens are only preserved as external moulds (PIMUZ 38652, 38,655, 39,488, 39,490, 39,492–39,494). Because of the poor preservation, we do not select these specimens as paratypes.

**Diagnosis**: As for genus, by monotypy.

**Description**: There are several specimens between bed 87–112 that are similar in cross section and apical angle, mostly preserved as external moulds. They range in the maximum lateral cross section from 6 to 20 mm and corresponding apical angles of 5–9°. The siphuncle is visible in only in the holotype, PIMUZ 39491, which also preserves a short part of the phragmocone. The phragmocone has a depressed cross section with a maximum lateral diameter of 8 mm and an expansion rate of 8.6°. It appears to have retrochoanitic septal necks and a relatively wide submarginal siphuncle (Fig. 3B). The septa are preserved as faint traces and are inclined towards the venter in anterior direction, with relative cameral length (RCL) of about 0.5. Further apicad, on the external mould, there are transverse lines, which could alternatively represent the septa, although they are more widely (> 1.0) and somewhat irregularly spaced. The preserved phragmocone is 12 mm long, while the entire specimen including external moulds is about 120 mm long.

**Remarks**: There are two potential traces of septa in the holotype, moderately long and inclined on the preserved phragmocone, and long and directly transverse on the apical part of the external mould. We think that the short, inclined traces are more likely to represent the septa, as they appear to be more regular and are preserved directly on the phragmocone. *Mojsisovicsteuthis boeckhi* (Stürzenbaum, 1875) has only a slightly higher apical angle, but as indicated by Mojsisivics (1882) and Hauer (1888), the siphuncle is situated on the narrower side of the cross section (see also Košťák et al., 2023), meaning that *M. boeckhi* has a compressed conch in contrast to the depressed *T. chuchichaeschtli*. Furthermore, the chambers are shorter in *M. boeckhi,* with only about 0.2 per conch diameter.

**Occurrence:** Ticino, Switzerland; Middle Triassic (Anisian).

Subclass **Orthoceratoidea** Teichert, 1967

Order **Pseudorthocerida** Flower & Caster, 1935

Family **Trematoceratidae** Zakharov, 1996

**Diagnosis**: Relatively small orthocones without any indication of conch curvature with the possible exception of the apicalmost parts. Expansion rate constant, around 5° with relatively little variation. Siphuncle central or very slightly subcentral, with straight or slightly expanded siphuncular segments and short ortho- or suborthochoanitic septal necks that lack the inner prismatic layer and have a reduced outer prismatic layer. Cameral deposits present, petal- or star-shaped. Endosiphuncular deposits parietal, possibly restricted to the septal necks and early ontogenetic stages. Embryonic shell where known comparatively large, up to 2 mm in diameter with blunt conical apex and cicatrix (modified after Zakharov, 1996).

**Included genera**: *Trematoceras* Eichwald, 1851; *Paratrematoceras* Schastlivceva, 1981; *Pseudotemperoceras* Schastlivceva, 1986. Doubtful: *Phatthalungoceras* Tongtherm & Nabhitabhata, 2018; *Zhuravlevia* Doguzhaeva, 1994.

**Remarks**: Several characters, such as the embryonic shell or the shape of the cameral deposits are known only from a limited number of taxa. However, due to the high consistency in other characters that are usually considered to be more variable between species (e.g., apical angle and shell sculpture), we assume that the variability in these lesser-known characters was likely low as well. Of course, new evidence for distinctly different morphologies may overturn our hypothesis but until then, we propose that the best approach is to classify all Mesozoic orthoceratoids within a single family. This includes the Early Cretaceous *Zhuravlevia* Doguzhaeva, 1994, although we include it here only tentatively due to the 90 million years gap between it and Triassic trematoceratids.

Like the Triassic orthoceratoids, there have been very few studies on Permian taxa. It is thus conceivable that the Trematoceratidae can be extended into the Permian. Nevertheless, the few available studies indicate that orthoceratoid diversity (or at least disparity) was comparatively higher during the Permian (see, e.g., Sweet, 1964; Niko et al., 2000; Yang et al., 2021; Niko & Ehiro, 2023). Thus, a possible extension of the Trematoceratidae to include Permian taxa must await a better phylogenetic understanding of the latter.

**Occurrence:** Global; Early–Late Triassic (Induan? Olenekian–Norian, Rhaetian?), Early Cretaceous? (Aptian?).

Genus ***Trematoceras*** Eichwald, 1851

**Type species**: *Orthocera elegans* Münster, 1841.

**Included species**: *T. elegans* (Münster, 1841); *T. boreale* Schastlivceva, 1986; *T. clarum* Schastlivceva, 1986; *T. hikichii* Niko et al., 2016; *T. insperatum* Schastlivceva, 1988; *T. lytosiphon* (Gemmellaro, 1904) comb. nov.; *T. mangishlakense* Schastlivceva, 1981; *T. solidum* Schastlivceva, 1988; *T. subcampanile* (Kiparisova, 1954); *T. vulgare* Schastlivceva, 1981; *T. watanabei* Niko & Ehiro, 2020.

**Emended diagnosis**: Trematoceratid with moderate to wide septal spacing and relatively wide, tubular, central siphuncles and orthochoanitic to suborthochoanitic septal necks with gradual septal neck transition.

**Remarks**: Differs from *Michelinoceras* by its shorter septal necks, a much larger protoconch with an initial chamber that is shorter than the subsequent chambers and a conical apex with a cicatrix. Additionally, the pronounced lamellar cameral deposits are unique to *Trematoceras*, producing star- or petal-shaped impressions. However, until the taphonomy, growth and formation of cameral deposits are better understood, the absence of this character should not be treated as diagnostic. *Paratrematoceras* differs mainly in having a narrower siphuncle with more sharply bent septal necks, while *Pseudotemperoceras* possesses expanded siphuncular segments and a tendency towards narrower cameral spacing.

Note that the above list of species includes only those with known internal structures. Many of the about 50 described species of Triassic orthoceratoids are only known from external characters and cannot be assigned to any genus with confidence and are considered nomina dubia. We also made only a minimal attempt to synonymise some of the previously described species, although we consider them more prevalent. “*Orthoceras*” *lytosiphon* Gemmellaro, 1904 from the Norian of Sicily is here reassigned to *Trematoceras*, as its internal characters appear to be virtually identical with the type species. We do not synonymise it with the latter species because we follow previously established or at least suspected synonymies. To further clarify the taxonomic status of the various *Trematoceras* species and other Triassic orthoceratoids, the original type specimens should be reinvestigated together with new material and analysed with quantitative morphometric methods.

**Occurrence:** Worldwide; Early–Late Triassic (Olenekian–Norian, Rhaetian?).

***Trematoceras elegans*** Münster, 1841.

**Fig. 9:**
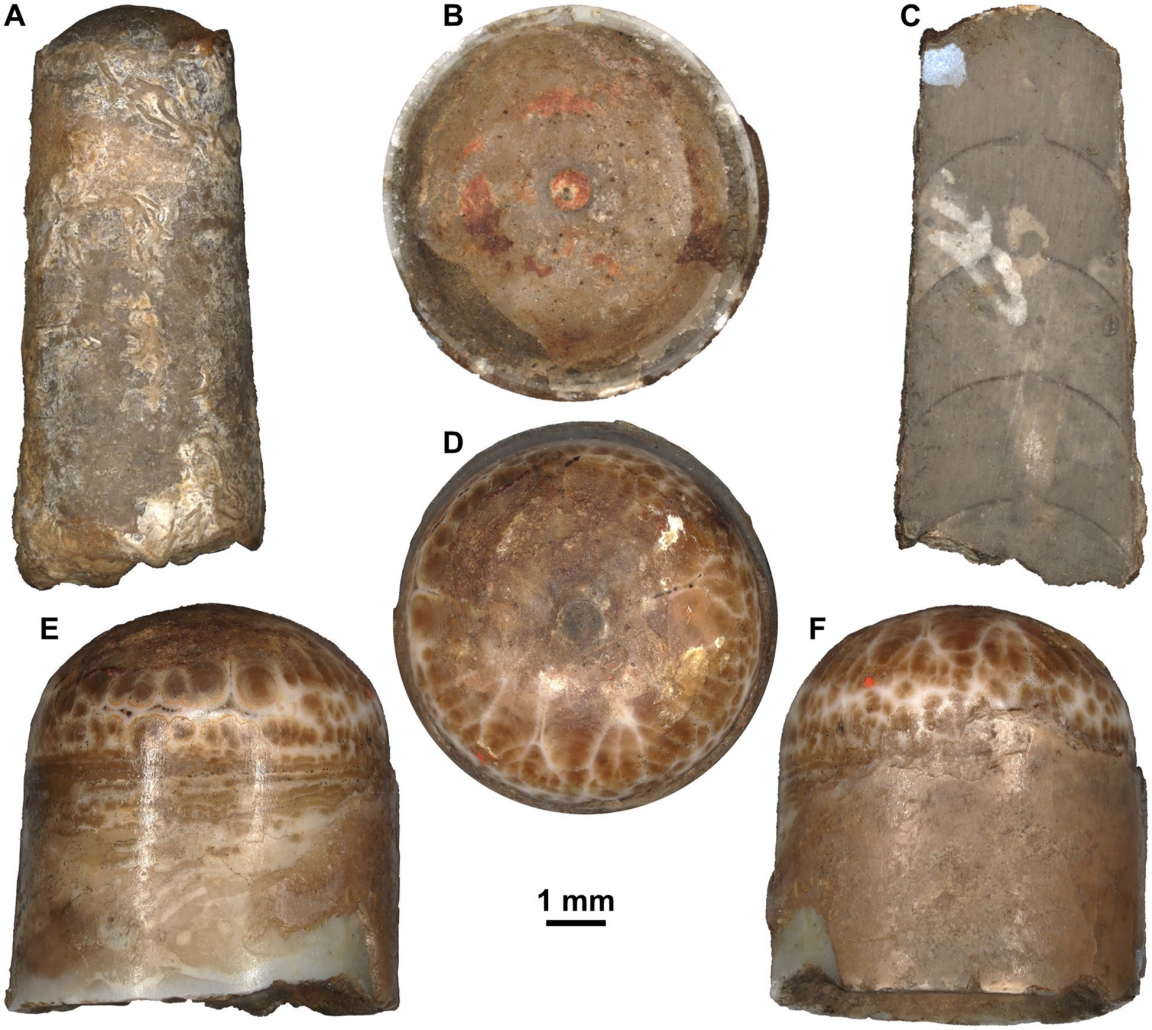
Lectotype and paralectotype of *Trematoceras elegans* (Münster, 1841) from the Carnian of the Cassian Formation. **A, C **SNSB-BSPG AS VII 1014, lectotype. **B, D–F** SNSB-BSPG AS VII 1015, paralectotype. **A** External (lateral?) view. **B** Apertural view. **C** Longitudinal section. **D** Apical view. **E** Lateral view, venter right. **F** Ventral view

**Fig. 10:**
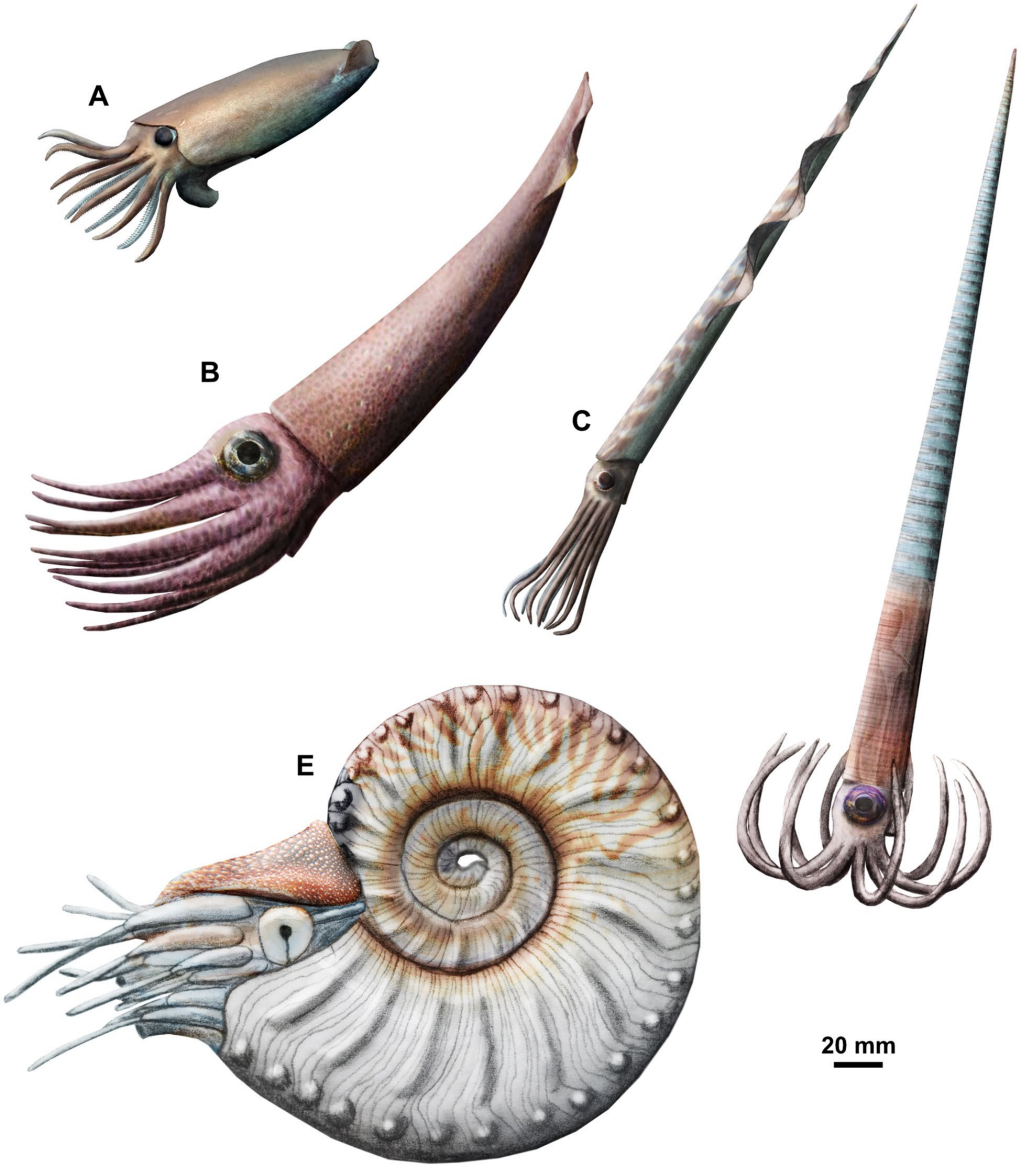
Reconstructions of non-ammonoid cephalopods from the Anisian of Monte San Giorgio. **A** *Breviconoteuthis breviconus* (Reis, 1907); **B** *Mojsisovicsteuthis* sp.; **C** *Ticinoteuthis chuchichaeschtli* gen. et sp. nov.; **D** *Trematoceras elegans* (Münster, 1841); **F** *Enoploceras rieberi* Pieroni, 2022

Figures 2A–H, 9A–F, 10D

1841*Orthocera elegans *Münster; p. 125; pl. 14, fig. 2a–c

1843*Orthocera Freieslebense *Klipstein; p. 143; pl. 9, fig. 4a–b

1843*Orthocera politum *Klipstein; p. 144; pl. 9, fig. 6

1847*Orthoceras dubium *Hauer; p. 260–261; pl. 7, fig. 3–8

1849*Orth. elegans *Münster–Quenstedt; p. 478–479; pl. 31, fig. 3–5

1851*Trematoceras elegans *Eichwald; p. 124; pl. 1, fig. 3

1859*Orthoceratites dubius *Hauer–Stoppani; p. 112; pl. 24, fig. 1–4

1867*Orthoceras *cf. *dubium *(Hauer)–Beyrich; p.138, pl. 3, fig. 3

1869*Orthoceras Campanile *Mojsisovics; p. 590

1869*Orthoceras elegans *Münster–Laube; p. 59; pl. 36, fig. 9

1869*Orthoceras politum *Klipstein–Laube; p. 60; pl. 36, fig. 8

1873*Orthoceras dubium *Hauer–Mojsisovics; p. 3–4; pl. 1, fig. 4, 5

1877*Orthoceras elegans *Münster–Barrande; Supplément, p. 65–66; pl. 483, fig. 4–15

1882*Orthoceras campanile *Mojsisovics–Mojsisovics; p. 291; pl. 93, fig. 1–4, 11

1882*Orthoceras elegans *Münster–Mojsisovics; p. 291; pl. 92, fig. 10–12

1882*Orthoceras politum *Klipstein–Mojsisovics; p. 293; pl. 92, fig. 13–14; pl. 93, fig. 7–8

1899*Orthoceras campanile *Mojsisovics–Tommasi; p. 16; pl. 2, fig. 1, 1a

1899*Orthoceras politum *Klipstein–Tommasi; p. 16; pl. 2, fig. 2, 2a

1907*Orthoceras campanile *Mojsisovics–Reis; p. 113–114; pl. 1, fig. 1

1952*Orthoceras elegans *Münster–Leonardi & Polo; p. 8; pl. 1, fig. 1, 4, 9, 11, 12; pl. 2, fig. 51–52

1964*Trematoceras *cf. *politum *(Klipstein)–Sweet; p. K229; fig. 156, 4a

1964*Trematoceras *cf. *elegans *(Münster)–Sweet; p. K229; fig. 156, 4b–c

1973*Michelinoceras campanile *(Mojsisovics)–Rieber; p. 71–72; pl. 17, fig. 11, 26–27

1991*Trematoceras elegans *(Münster)–Bizzarini & Gnoli; p. 112; pl. 1, fig. 1–4; pl. 2, fig. 1–2

**Lectotype:** Despite the long research history and its status as type species of *Trematoceras*, a lectotype was never designated for *T. elegans*. Münster (1841) mentioned that the species was not rare in the Cassian Formation, but it is not clear how many specimens he had at his disposal, though he illustrated three specimens that can be considered as syntypes. Only the specimen on his pl. 14, fig 2c (SNSB-BSPG AS VII 1014) has been sectioned to show the structure of the septal necks (Fig. 9A, C). This specimen is thus best suited as lectotype and is here designated as such. Nevertheless, the specimen is strongly corroded and does not show cameral deposits. These are clearly visible in specimen SNSB-BSPG AS VII 1015, figured on pl. 14, fig 2b in Münster (1841), which we designate as paralectotype (Fig. 9B, D–F). We do not designate the other specimens in Münster’s collection as paralectotypes, as their internal characters are unknown and accordingly, there is a small chance that these specimens represent separate species. Nevertheless, we think that it is likely that there is only a single species in the Cassian Formation.

**Diagnosis**: *Trematoceras* with expansion rate (ER) of about 5°, relative siphuncular size (RSS) of approximately 0.1 and relative cameral length (RCL) between 0.5 and 1.5. Septal necks very short, constricting siphuncle slightly at septal foramen. Lamellar cameral deposits present, arranged radially in petal-shaped sectors. Embryonic shell relatively large, up to 2 mm in diameter compared to an adult size of at least 25 mm, possibly up to 50 mm (poor data). Endosiphuncular deposits unknown.

**Material**: 16 specimens from the Besano Formation are comfortably assigned to *Trematoceras*, e.g., due to visibly central siphuncle. Two specimens were sectioned, one of them revealing septal necks, confirming the species assignment. The rest of the material was assumed to represent the same species due to the general low morphological variability. Further 19 specimens were only poorly preserved (e.g., as external moulds) but agree in cross section and expansion rate and thus, an assignment to *Trematoceras elegans* is assumed. In addition to the three specimens (PIMUZ 3770, 3781, 3782) already illustrated by Rieber (1973), we include PIMUZ 30979, 38,255, 38,348, 38,625, 38,651–38,654, 38,669, 38,693, 38,966, 38,993, 39,021, 39,024–39030, 39,056–39065, 39,486, 39,587. Furthermore, PIMUZ 29943 is noted as loaned to the Museo dei fossili del Monte San Giorgio, Meride (CH) and was not investigated. In total, this adds up to 36 specimens from the upper Lower and middle Besano Formation. Stratigraphic distribution of the material: bed 41 (PIMUZ 29943, 39,056, 39,586), bed 45 (PIMUZ 3781, 39,486), bed 47 (PIMUZ 3782, 30,979), bed 49 (39,587), bed 58 (39,057, 39,058), bed 61 (PIMUZ 3770, 39,024–39030, 39,065), bed 69 (PIMUZ 38966, 38,993), bed 72 (PIMUZ 38255, 38,348, 38,625), bed 74 (PIMUZ 39021), bed 83, (PIMUZ 38693), bed 88 (PIMUZ 39059), bed 94 (PIMUZ 39060), bed 98 (PIMUZ 38651–38,654), bed 100 (PIMUZ 38669), bed 104 (PIMUZ 39061, 39,062), bed 148 (PIMUZ 39063), bed 162 (PIMUZ 39064).

**Description**: The specimens from Monte San Giorgio are either preserved as internal moulds of phragmocones or body chambers, or as external moulds, leaving essentially a hollow tube in the matrix. The conch is virtually straight with circular cross section. The apertural diameter of the fragments is on average 14.0 mm. The largest specimens, PIMUZ 38348 and 38625 (both from bed 72), have an apertural diameter of 25 mm, while the smallest specimen, PIMUZ 38693 from bed 83, reaches from 2 to 4 mm. External moulds are usually preserved over a considerable length up to 183 mm (PIMUZ 3782, bed 47; see Rieber, 1973), while internal moulds are usually broken into shorter fragments. The expansion rate remains constant within the same specimen, on average 4.9°.

**Remarks**: Rieber (1973) considered only the species “*Michelinoceras*” *dubium* (Hauer, 1847) and “*M*.” *campanile* as possible candidates for the material from the Besano Formation (both differing only in the length of the body chamber and septal spacing) and assigned all material to the latter species. Because he did not section any specimens, his taxonomic identifications are uncertain, as Schastlivceva (1981, 1986, 1988) showed that internal characters set apart the genera. The specimen sectioned here (PIMUZ 39586, Fig. 2D, E) confirms an assignment to *Trematoceras*. Due to the poor data on most species of *Trematoceras*, it is challenging to differentiate between the about 50 species of Triassic orthoceratoids that have been established to date. Internal characters are missing entirely for many Triassic orthoceratoids (listed under “*Orthoceras*” in Table 1), and intraspecific variation has typically not been investigated. As the *Trematoceras* morphotype seems to represent the majority of the known trematoceratids, it is likely that many of them belong to this genus (Schastlivceva, 1988). However, at the same time it is also likely that many species represent junior synonyms. Externally, they are all very similar and it is doubtful whether slight variation in the spacing or coarseness of the growth lines justify species separation. It is probable that cameral and endosiphuncular deposits provide additional insights, but knowledge on them is very patchy. There may also be some variation in the length and shape of the septal necks, but they have similarly rarely been investigated. It is furthermore problematic that many species are only available as drawings in old monographs and the original type material has never been photographed and published (as exemplary shown for the type species). These species are thus mostly known from short descriptions that are insufficient from a modern taxonomic perspective. Indeed, it is quite possible that some type specimens will prove to be difficult to find or are lost.

*T. elegans* has been differentiated from other species by its apical angle, the length of its body chamber, cameral spacing, or details in the ornamentation. However, as elaborated above, the variation of most of these characters is so poorly known that the utility of these characters in differentiating between species is doubtful. Already Quenstedt (1849) was of the opinion that *“Orthocera” freieslebenense* Klipstein, 1843 was identical with *T. elegans*, as according to him, it was based on taphonomic features. This opinion appears to have been accepted by others, as the species has not been studied since then. According to Quenstedt (1849), the species *O. ellipticus* Klipstein, 1843 (preoccupied by *O. ellipticus* Münster, 1840, thus replaced by *O. subellipticus* d’Orbigny, 1850), characterised by an elliptical cross section, represents deformed body chambers of *T. elegans*, although other authors treated *O. subellipticus* as valid. Later, Mojsisovics (1882) indicated that *T. elegans* and *T. campanile* Mojsisovics, 1869 differ only in the smaller size of the latter and that intermediate specimens would invalidate the latter species. This was corroborated by Leonardi and Polo (1952), who reported intermediately sized trematoceratids from the Cassian Formation, arguing that the two species cannot be reliably separated. The trematoceratids from the Besano Formation appear to be closer in size to other Anisian specimens. However, according to Quenstedt (1849), specimens with a “7/4 inch diameter” (= ca. 4–5 cm) occur as well in the Cassian Formation. Similar sizes have been reported from slightly younger orthoceratoids from Austria by Hauer (1847). Generally, the size data is so poor that it does not allow for a species separation.

We cannot find any convincing argument for splitting the alpine species of *Trematoceras* into more than one species. While we do not rule out the possibility of several sympatric species, the current species do not reflect natural units, because they were defined based on features that are either known to be variable within the same locality or even within a single specimen, or else are unknown in a lot of specimens. Conversely, characters that might carry some diagnostic value such as those of the siphuncle are poorly known. Therefore, in our opinion, the best approach is to provisionally accept only the type species, *T. elegans*, as valid until more data becomes available. Species not included in the synonymy list are therefore regarded as nomina dubia (compare species listed as “*Orthoceras*” in Table 3). Norian and Rhaetian orthoceratoids from the alps are poorly known for the most part and currently, we cannot confirm their assignment to *T. elegans*. *Trematoceras* cf. *triadicum* described by Jeletzky and Zapfe (1967) appears to have slightly longer septal necks and a wider siphuncle, but the limited data make it impossible to judge whether this is within the range of intraspecific variation. Of the alpine orthoceratoids where internal characters are known, only “*Orthoceras*” *salinarium* Hauer, 1846 appears to differ in a narrower siphuncle, which is here assigned to *Paratrematoceras* (see below).

**Occurrence:** Alps, Europe; Middle–Late Triassic (Anisian–Carnian).

Genus ***Paratrematoceras*** Schastlivceva, 1981

**Diagnosis**: Trematoceratid with narrow, tubular siphuncle with short orthochoanitic septal necks that sharply bend in adapically at the transition to the septum.

**Type species:** *P. shevyrevi* Schastlivceva, 1981

**Included species**: *P. ornatum* Schastlivceva, 1981; *P. salinarium* (Hauer, 1846) comb. nov.

**Remarks**: Differs from *Trematoceras* in its narrower siphuncle with sharply bent, orthochoanitic septal necks.

In addition to the species listed by Schastlivceva (1988), which includes specimens described as *Trematoceras* aff. *dubium* from the Olenekian of Afghanistan (Kummel & Erben, 1968), *Orthoceras salinarium* Hauer, 1846 from the Norian of Austria may be referred to the genus, as it has a similarly narrow siphuncle with sharply bent septal necks. However, this is purely based on Hauer’s (1846) drawings and investigation of his type material is required to test this assignment. *Michelinoceras* sp. A in Tongtherm et al. (2016) from the Anisian of Thailand possibly belongs to *Paratrematoceras* due to its narrow siphuncle and orthochoanitic septal necks. Several species from Olenekian to Anisian deposits have been assigned to *Paratrematoceras* by Grădinaru et al. (2007), but since they were not accompanied by illustrations, they represent nomina nuda and the presence of the genus in these rocks is unconfirmed. Consequently, the genus appears to be already present and relatively widespread in the early Middle Triassic, although further studies are needed to differentiate it from *Trematoceras* with certainty.

**Occurrence:** Northern Caucasus, Alps?, Thailand?; Middle Triassic (Anisian)?, Late Triassic (Norian).

Genus ***Pseudotemperoceras*** Schastlivceva, 1986

**Diagnosis**: Trematoceratid with narrow septal spacing and relatively wide siphuncle with expanded segments and suborthochoanitic septal necks.

**Type species**: *Pseudotemperoceras pulchrum* Schastlivceva, 1986

**Included species**: *P. nyalamense* (Chen, 1981) comb. n.

**Remarks**: This genus is easily distinguishable from other trematoceratids by its expanded siphuncular segments with suborthochoanitic septal necks and its relatively narrow septal spacing. In addition to the type species, we assign *Michelinoceras nyalamense* Chen, 1981 from the Induan–Olenekian transition of Tibet to the genus, which is very similar to the type species except for its more widely spaced septa. The same species was also reported from the Anisian of Guizhou, South China, although without figures (Stiller, 2001). We also suspect that the Anisian *Phatthalungoceras* Tongtherm & Nabhitabhata, 2018 is a junior synonym of *Pseudotemperoceras* (see below), in which case *Phatthalungoceras srisuki* Tongtherm & Nabhitabhata, 2018 would constitute a third species. Although the genus has not been documented outside of present-day Asia, tectonic reconstructions indicate that the genus had a very wide distribution during the Early Triassic (compare Fig. 8A).

**Occurrence**: Siberia, Himalaya, South China? Early Triassic (Induan?, Olenekian), Middle Triassic (Anisian)?

Genus ***Phatthalungoceras*** Tongtherm & Nabhitabhata, 2018

**Diagnosis**: Trematoceratid with depressed cross section, short chambers and expanded siphuncular segments.

**Type species**: *Phatthalungoceras srisuki* Tongtherm & Nabhitabhata, 2018

**Included species**: Only the type species.

**Remarks**: The only known specimen was first described as *Tienoceras* sp. A in Tongtherm et al. (2016) and later established as new species and genus by Tongtherm and Nabhitabhata (2018). However, the holotype is relatively poorly preserved, and judging by the illustrations, it is possible that the reported depressed cross section is a result of compaction or erosion. If this is the case, the genus is essentially identical with *Pseudotemperoceras* due to its short chambers and expanded siphuncular segments.

**Occurrence**: Thailand; Middle Triassic (Anisian).

Genus ***Zhuravlevia*** Doguzhaeva, 1994

**Diagnosis**: Small orthocone with circular cross section, siphuncle central with short suborthochoanitic septal necks and slightly expanded siphuncular segments, septal spacing relatively wide, cameral and endosiphuncular deposits unknown.

**Type species**: *Zhuravlevia insperata* Doguzhaeva, 1994

**Included species**: Only the type species.

**Remarks**: *Zhuravlevia* resembles *Pseudotemperoceras* but differs mainly in its more widely spaced septa. Its isolated occurrence after a hiatus of about 90 million years is curious and needs to be confirmed by additional material to exclude it as a result of reworking. Nevertheless, the genus is similar to other trematoceratids and likely belongs to the same lineage.

**Occurrence**: Caucasus; Cretaceous (Aptian).

## Data Availability

All material newly described here is housed in the collection of the Palaeontological Institute and Museum of the University of Zurich (PIMUZ).
